# A systematic review of the use of burden of treatment theory

**DOI:** 10.1177/26335565251314828

**Published:** 2025-05-09

**Authors:** Rachel C Smyth, Georgia Smith, Emily Alexander, Carl R May, Frances S Mair, Katie I Gallacher

**Affiliations:** 1 3526 University of Glasgow, Glasgow, UK; 2 156606 London School of Hygiene and Tropical Medicine Faculty of Public Health and Policy, London, UK

**Keywords:** systematic review, burden of treatment, qualitative

## Abstract

**Background:**

Treatment burden describes the workload undertaken by people with chronic illness and multimorbidity to manage their healthcare demands and the impact on their wellbeing. Burden of Treatment Theory (BOTT) describes the work that people with multimorbidity do to self-manage chronic illness/multimorbidity and the factors that affect capacity (personal and healthcare resources, support network) to meet treatment demands. Here we aim to identify and characterise the different applications of Burden of Treatment Theory in research; to explore the contribution of Burden of Treatment Theory to advancing knowledge and understanding of treatment burden and capacity issues and to identify critiques or limitations of Burden of Treatment Theory in research.

**Methods:**

Systematic review of BOTT research published in the English language. Databases searched were Web of Science, Scopus, Medline, CINAHL and medRxiv.org. We also consulted with experts in the field. Two reviewers screened titles, abstracts and papers and undertook data extraction. Quality appraisal was undertaken using adapted CASP checklists for qualitative studies and systematic reviews and a Mixed Studies Review checklist.

**Results:**

Thirty papers included: 16 qualitative studies; 5 systematic reviews; 3 protocols; 3 discussion papers, a theory conceptual paper, a realist review and a feasibility trial. Most (n=17) originated in UK, with 3 from Australia and Argentina, 2 from Norway and one each from United States and Malawi. Nine papers mentioned use of BOTT constructs but 21 additionally provided rationale for BOTT use and demonstrated engagement with the theory. Two papers adapted/refined BOTT to the context of their research focus. Twenty-seven studies prospectively outlined use of BOTT, with only 3 applying BOTT retrospectively to report study outputs and ‘inform analysis’ of findings.

**Conclusion:**

BOTT provides a useful conceptual, analytical and sensitising lens in studies focusing on both the characterisation and alleviation of treatment burden through healthcare interventions, and the constructs discussed are stable and applicable across multiple settings. Future research could include use by empirical researchers in contexts needing more adaptation and critical assessment.

## Introduction

### Understanding treatment burden

Treatment burden describes the workload undertaken by people with long-term conditions to manage the demands of their healthcare and the impact of that workload on wellbeing.^1,2^ Individuals and their support networks devote actions and resources to health management that can accumulate as a considerable healthcare workload.^3^ That workload can create a perceived burden for the individual and their supporters, which may result in disengagement from health services or poor quality of life.^4^

The wider environmental contexts of social support, structural inequality and resource accessibility contribute to the workload of a growing population of older and/or multimorbid patients who face years of long-term illness management.^5^ Crucial qualitative research conducted over the past decade has advanced the understanding of treatment burden in the context of multimorbidity and the increasing delegation of health-related tasks from healthcare systems to the people living with chronic illness.^2,3,5–10^ Implementation theories, especially Normalisation Process Theory (NPT), have facilitated exploration of the ways that treatment work becomes embedded in patients’ routines.^6–8^ NPT is therefore useful for research that aims to understand the tasks and processes followed by patients to maintain and improve their health, in a range of clinical settings and contexts.^15,16^ Alongside consideration of workload, it is important to acknowledge that the ability to handle workload varies between individuals depending on a range of factors, and the Theory of Patient Capacity has helped to conceptualise these factors including the reshaping of biography, available resources, the environment, the realisation of work, and social support.^9^ Theory of Patient Capacity is therefore useful for research that aims to examine the factors that affect a person’s ability to manage their health. The Cumulative Complexity Model (CCM) has also proved useful in its conceptualisation of the delicate balance between workload demands and patient capacity to manage those demands, the latter depending on a variety of physical, psychosocial and contextual factors.^11^ Research that aims to understand the interplay between workload and capacity may utilise CCM to underpin their methods and explore these relationships. Burden of Treatment Theory further expands on the intersecting concepts of healthcare workload for the modern-day individual with multimorbidity and their capacity to manage that workload.^12^

### What is burden of treatment theory?

**Figure 1.**
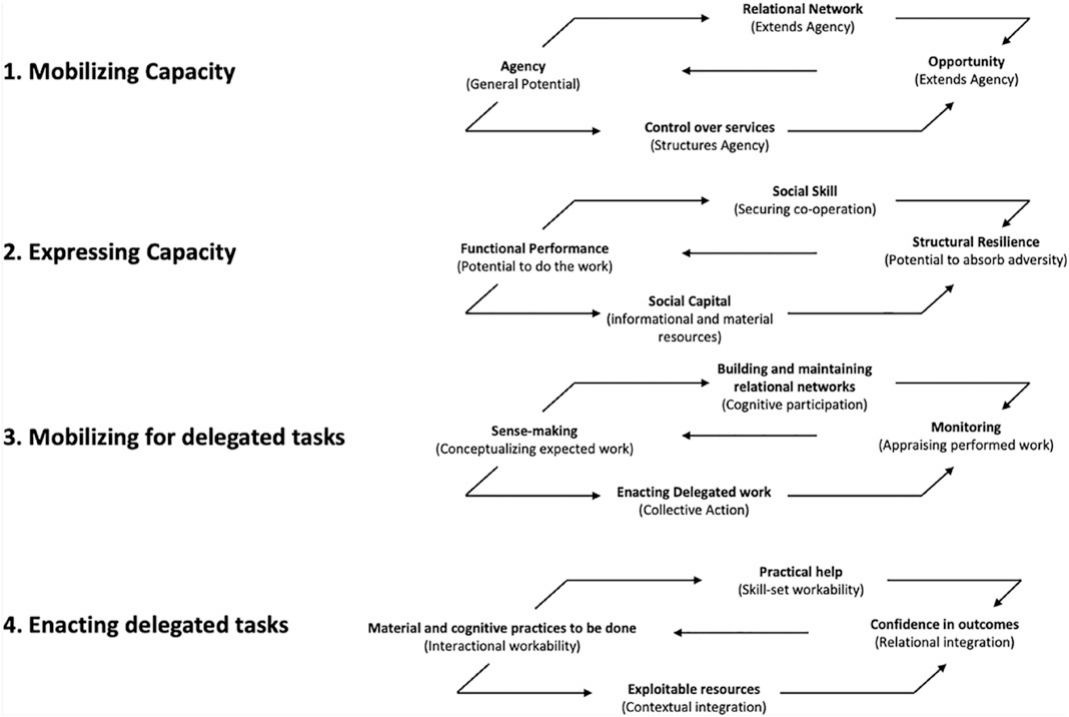
A conceptual map of the Burden of Treatment Theory outlined by Chikumbu et al.^14^

Burden of Treatment Theory (BOTT) identifies, characterises and explains the social mechanisms that motivate and shape patients’ and caregivers’ effective participation in their care. It facilitates understanding of these lived experiences in terms of patient and caregiver work that is delegated by healthcare systems.^12^ Building on earlier work that focused on the taxonomy of treatment burden and patient capacity,^2,6,11^ BOTT outlines how patient capacity is influenced by structural and contextual factors that extend beyond the individual, and highlights the importance of understanding these interactions to mitigate treatment burden- particularly in the new era of patient-hood characterised by multimorbidity and a focus on self-management.^13^ The theory outlines factors which underpin the mobilisation and expression of capacity (how patients use their available social and psychological resources to engage with their healthcare) in order to illustrate the importance of structural and social support as well as accessibility to reduce treatment burden for patients. Figure 1 illustrates a conceptual map of Burden of Treatment Theory.^14^

### The purpose of this review

To our knowledge, this is the first systematic review of Burden of Treatment Theory. Previous reviews of theories such as NPT have been instrumental in highlighting researcher responses to the theory, assessing theoretical understanding and application, and determining the contribution of the theory to clinical practice.^15,16^ Since 2014, Burden of Treatment Theory (BOTT) has been used in a diverse range of studies and it is therefore the opportune time to review how it has been applied in research, and to what extent its application has contributed to advancing our knowledge of treatment burden and capacity issues. This will aid in directing future BOTT work in order to maximise the potential contribution and utilisation of the theory in understanding treatment burden, which is a particularly important issue in those with multimorbidity.

The aims of this review are:

• To identify and characterise the different applications of Burden of Treatment Theory in research
• To explore the contribution of Burden of Treatment Theory to advancing knowledge and understanding of treatment burden and capacity issues
• To identify critiques or limitations of Burden of Treatment Theory in research

## Methods

A systematic review of BOTT research published in the English language was conducted. The review was registered on PROSPERO, the International Prospective Register of Systematic Reviews (CRD42022308416, https://www.crd.york.ac.uk/prospero/display_record.php?RecordID=308416).

### Systematic searches

Prior to the systematic review, a scoping search was conducted using Google Scholar to find key papers relevant to the search. Our search strategy focused on identifying studies that cited the original paper presenting BOTT,^12^ which was published in June 2014, therefore this date was set as a limit. The databases searched were: Web of Science, Scopus, Medline, CINAHL and medRxiv.org. The search was undertaken in December 2021 and updated in June 2022. Our full search strategy is available in the Supplemental Material file. Due to lack of funding for translation, English language papers only were included.

Original research studies, conference papers, systematic reviews, theory or conceptual discussion papers, protocols, conference papers or pre-prints published in the English language after June 2014 that cited the original Burden of Treatment Theory conceptual paper^12^ and furthermore engaged with, applied or discussed the theory were included. Our outlined examples of theory engagement included: use in data analysis or collection, to guide or inform interview methods, to thematise or characterise data or discussions, to inform methods of intervention development, or any other outlined application. There were no restrictions on methodology or the type of study design eligible for inclusion, as the primary focus of this review was to characterise applications of BOTT. Editorials, letters, conference abstracts, theses or dissertations were excluded, as were those papers which made only passing reference to BOTT and did not engage with, apply or discuss the theory.

### Screening

Records identified through database searches were downloaded onto Endnote reference manager software where duplicates were removed, and all references were then uploaded onto DistillerSR. All screening was performed using DistillerSR software by two independent reviewers who had not been involved in the development of BOTT, with two additional reviewers assessing papers that required a second opinion.

### Quality appraisal

Quality appraisal was carried out using adapted CASP checklists^17^ for qualitative studies and systematic reviews and a Mixed Studies Review checklist^18^ for mixed methods studies. Second and third reviewers independently appraised the quality of included studies. The purpose of quality appraisal was to inform understanding of the quality of the literature, and so no studies were to be excluded based on results of quality appraisal.

### Data extraction

A data extraction form (see Table 1) was designed and completed using DistillerSR software and all papers were double-reviewed. Data extraction included characteristics of included papers such as country of origin, methodology, aims and outcomes, as well as each paper’s application, discussion and commentary of BOTT. We extracted information from aims, methods, results and discussion sections of included papers.

**Table 1.** A summary of the data extraction form that was designed on DistillerSR.

Data extraction form
1. Author
2. Title
3. Year
4. Country
5. Study type
6. Participant demographics (age, sex, ethnicity, sample size, key inclusion criteria, socioeconomic status)
7. Study setting
8. What was the research question and focus area?
9. What were the aims and objectives of the study?
10. How was BOTT used? • To underpin data analysis or collection • To guide or inform interview methods • To inform methods of intervention development • To thematise or characterise data • Other: ________ ** Details:**
11. Was BOTT applied prospectively or retrospectively? Yes/no ** Details:**
12. Did the authors provide a rationale for using BOTT? Yes/no ** Details:**
13. Did the authors use BOTT in combination with other theories? Yes/no ** Details:**
14. Did the authors compare BOTT with other theories? Yes/no **Details:**
15. Was BOTT refined, extended or adapted in any way? Yes/no ** Details:**
16. Does the paper discuss BOTT constructs and their operationalisation? Yes/no ** Details:**
17. Did the paper comment on the benefits, adaptability or usefulness of BOTT? Yes/no ** Details:**
18. Did the paper identify any limitations or problems associated with BOTT? Yes/no ** Details:**
19. Does the paper exclusively use BOTT descriptively to thematise/characterise the data? Yes/no ** Details:**
20. Is the claimed use of BOTT evident in the paper? Yes/no **Details:**
21. What were the overall outcomes and conclusions of the paper?
22. How, if at all, has BOTT contributed to the outcomes and conclusions of the paper?

### Data analysis

Initial interpretation work was undertaken, and descriptive tables created to outline the context, aims, objectives, outcomes and conclusions of included studies, as well as their methodology and application of BOTT. Inductive analysis was undertaken to identify key themes from the articles, and the application of BOTT was assessed in different study types to provide understanding of the current and future use of BOTT in clinical and non-clinical research. Discussion of the thematic constructs of BOTT and the level and nature of engagement and critique of BOTT between studies were appraised to characterise theory use fully and assess the contribution of BOTT to the understanding of treatment burden and capacity issues.

## Results

### Search results

**Figure 2.**
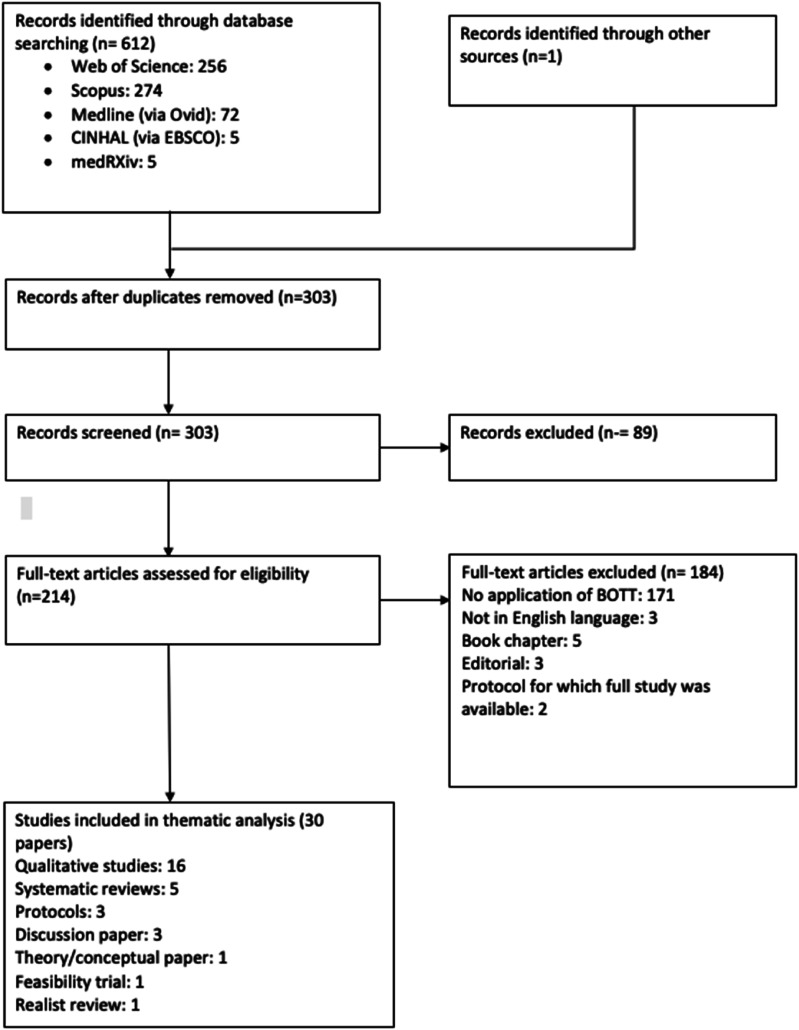
PRISMA flowchart showing the study selection process of this review.

Searches yielded 613 citations. Figure 2 illustrates that after the removal of 310 duplicates, 303 papers remained and were screened as titles and abstracts, with 89 of these papers excluded due to not meeting inclusion criteria. 214 papers then underwent full-text screening and 184 were excluded. In total, 30 papers met inclusion criteria for this review. All included studies, assessed via quality appraisal checklists,^17,18^ were of high quality and a table outlining appraisal and quality scoring of the included papers is available in Supplemental Materials provided.

### Types of studies

As displayed in Table 2, 30 papers applied BOTT across a range of different study types: sixteen qualitative studies,^14,19,23,24,27–29,33–35,37,39,40,43,45,46^ five systematic reviews,^21,32,36,41,44^ 3 protocols,^25,26,38^ three discussion papers,^22,31,47^ a theory conceptual paper,^30^ a realist review^42^ and a feasibility trial.^20^ The majority (n=17) of papers originated in the UK, the country of origin of BOTT, with others from Australia,^22,43,47^ Norway,^39,40^ Argentina,^44–46^ the United States^19^ and Malawi.^14^ Three systematic reviews focused on broad ranges of studies from Europe, North America, Asia, Africa and Australia.^21,32,36^ Most (n=18) included papers listed authors who were also authors of the original BOTT paper.^14,19–21,24,26,27,29,30,34,36–38,41,44–47^

**Table 2.** Author, year, country, paper type, aims and research focus, setting, methodology and outcomes of included papers.

First author, year	Country	Paper type	Aims and research focus of paper	Paper setting and methodology	Outcomes/Conclusions
Abu Dabrh et al, 2021^19^	United States	Qualitative study	To observe and assess concordant and discordant elements between the minimally disruptive medicine (MDM care model) and HIV care clinic model	Community setting Semi-guided interviews and qualitative case study discussion	The HIV clinic care model is aligned with the MDM model of care through support of patient capacity and abilities to minimise patient workload
Ainsworth et al, 2019^20^	UK	Feasibility trial	To assess feasibility of an RCT to determine the acceptability of an asthma self-management digital intervention, “My breathing Matters”	Community setting Questionnaires assessed patient burden	A full-size confirmatory trial to assess the effectiveness of ‘My breathing Matters’ is feasible and acceptable
Austin et al, 2021^21^	North America, Asia, Europe, Africa	Systematic review	To systematically review the qualitative literature on lived experiences of CHF to identify, characterise and explain interactions between symptoms and burden of treatment in chronic heart failure	Community and hospital settings Systematic review	Symptoms are integral to the patient experience of CHF and burden of treatment, and they impede patient efforts to engage in self-care- symptoms increase patient workload
Brunelli et al, 2021^22^	Australia	Case study discussion	To discuss the impact of rapid service changes due to COVID-19 on individuals with disabilities, and to assess the role of disability nurse navigators	Community setting Case study discussion	Telehealth has attempted to enable continuity, yet has potentially exacerbated the workload of patients with disabilities during COVID-19 – patients who are also suffering an increased burden due to impoverished social networks in the context of the pandemic
Callan et al, 2021^23^	UK	Qualitative study	To explore the lived experience of ‘brain fog’ due to COVID-19 and how to support patients	Community setting. Qualitative interviews	Experiences are diverse and varied, and healthcare for these patients should include an ongoing therapeutic relationship with a clinician and accessible services
Chikumbu et al, 2022^14^	Malawi	Qualitative study	To investigate the experiences and treatment burden for patients with multimorbidity in urban and rural Malawi, and to assess how useful NPT and BOTT are to structure accounts of these experiences	Community (LMIC- both urban and rural settings). Semi-structured in-depth interviews	Experiences of multimorbidity in Malawi (LMIC) can be illustrated successfully using BOTT/NPT- however, ‘lack’ of access to treatments or services adds an important dimension to the concept of treatment burden and could be integrated into future conceptual research
Corbett et al, 2020^24^	UK	Qualitative study	To provide a clear description of how the intervention tool CHAT&PLAN was optimised and planned and how findings shaped expectations of participants	Community setting. Qualitative interviews	Elicited barriers and facilitators to implementation and illustrated a need for training to deliver CHAT&PLAN in practice
Early et al, 2018^25^	UK	Protocol for a mixed methods study	To understand challenges to referral and uptake of pulmonary rehabilitation in primary care in the UK	Community setting. Protocol – mixed methods study (qualitative interviews)	N/A
Foster et al, 2019^26^	UK	Protocol for a cohort study	To assess the impact of cancer diagnosis and treatment on patient workload and health outcomes	Hospital setting. Protocol – cohort study (patient questionnaires)	N/A
Gilbert et al, 2021^27^	UK	Qualitative study	To characterise and explain factors influencing patient preference for virtual consultations in an orthopaedic rehabilitation setting	Hospital and community settings. Qualitative semi-structured interviews	Key factors identified which influence patient preferences for virtual consultation, and a conceptual model of these factors produced
Green et al, 2016^28^	UK	Qualitative study	To examine the perspectives of palliative care patients on treatment burden and use of the emergency department	Hospital setting. Qualitative semi-structured interviews	Participants felt local services were complex and inconsistent, and were more likely to attend ED due to work required to make sense of alternative options
Hounkpatin et al, 2020^29^	UK	Qualitative study	To explore patients’ and kidney care teams’ perspectives on treatment burden and factors supporting capacity for older adults with CKD	Community setting. Qualitative semi-structured interviews	Patients felt that provision of CKD information was poor and that there was a lack of control over treatment choice. Additional issues included loss of social life and employment. Improved understanding and measures needed to reduce treatment burden of CKD.
Hunt et al, 2017^30^	UK	Theory discussion paper	To set out and design a theory of the behavioural and social mechanisms through which people balance capacity and accountability	Community setting. Theoretical discussion paper, theory design	Outlined cognitive authority theory to assist in explaining how patients manage relational aspects of inequalities in power and expertise
Husebo et al, 2019^31^	Norway	Discussion paper	To present and discuss a nurse Assisted eHealth service for patients with non-communicable diseases of heart failure and colorectal cancer	Community setting. Case study discussion	Presented and explained eHealth solution and illustrated a new theoretical model of healthcare solutions post-hospital discharge – the proposed ‘BOT-app’
Jakubowski et al, 2022^32^	North America, Asia, Australia, Europe	Systematic review	To assess the current behaviours and attitudes among pregnant women regarding the self-management and self-monitoring of chronic conditions in pregnancy	Community and hospital settings. Systematic review	The primary motivational factor for women to self-manage their conditions is the health of their baby. The effectiveness of self-management is impacted by their support networks and their understanding of their condition
Knowles et al, 2017^33^	UK	Qualitative study	To co-design (with patients and professionals) new interventions to improve safety for patients with multi-morbidities in primary care, and to assess the acceptability and feasibility of these approaches	Community setting. Qualitative workshops and focus groups	Health professionals and patients have shared visions for improving primary care for patients, and bringing both together to co-design safety ideas is useful for workload alleviation
Kyle et al, 2020^34^	UK	Qualitative study	To examine potential barriers and enablers for minimising treatment burden and maximising patient capacity for those affected by stroke	Community and hospital (primary and secondary stroke services). Qualitative semi-structured interviews	The importance of patient-centred care in delivery of stroke services was highlighted and is vital to alleviate the treatment burden on stroke patients
Ladds et al, 2020^35^	UK	Qualitative study	To document patients’ lived experiences of ‘long covid’ and assess service provision and potential improvements	Community setting. Qualitative semi- structured interviews and focus groups	The patient experience of ‘long covid’ is varied, burdensome and uncertain and more service support is required to meet patient needs
Lippiett et al, 2018^36^	Europe, north America, Australia	Systematic review	To characterise and explain treatment burden features in relation to patients living with lung cancer or COPD	Healthcare systems (primary and secondary) in Europe, north America and Australia. Systematic review	There are significant differences in treatment workload between lung cancer and COPD, and workload exceeding capacity is a driving factor of treatment burden
Lippiett et al, 2022^37^	UK	Qualitative study	To identify, characterise and explain treatment burden in COPD and lung cancer	Hospital setting. Qualitative methods, semi-structured interviews, non-participant observation	The diagnostic process is important for treatment burden; biographical disruption or erosion impacts on patient experiences
May et al, 2015^38^	UK	Protocol for qualitative meta-synthesis and conceptual modelling study	To characterise and explain patient journeys through care in CKD, chronic heart failure and COPD and build a conceptual model	Community and hospital settings. Protocol for conceptual modelling study, qualitative meta-synthesis	N/A
Nordfonn et al, 2019^39^	Norway	Qualitative study	To explore chronic heart failure patients’ perceptions of treatment burden	Hospital setting- heart failure outpatient clinic. Individual semi-structured qualitative interviews	Heart failure treatment burden constitutes many self-care and emotional burden challenges for patients
Nordfonn et al, 2020^40^	Norway	Qualitative study	To explore how patients with heart failure perceive their capacity to manage treatment and self-care	Hospital setting- heart failure outpatient clinic. Individual semi-structured qualitative interviews	Identified important elements for heart failure patients which enhance their capacity for treatment and self-care and highlights role of healthcare professionals in providing support
O’Connor et al, 2016^41^	UK	Systematic review	To identify and synthesise literature on barriers and facilitators to engagement with digital health interventions	Community setting. Systematic review	There are many interconnecting factors affecting engagement and future research should address gaps in the knowledge, particularly focusing on enrolment strategies, accessibility and investment in computer literacy
Papoutsi et al, 2019^42^	UK	Realist review	To explore how group clinics can provide support for young people with type 1 and 2 diabetes	Community setting. Realist review	To engage people in group clinics, it is important that they emphasise self-management, promote affinity between patients, are safe, and balance group and individual needs
Quigley et al, 2021^43^	Australia	Qualitative study	To explore how systemic complexity and associated work is experienced by carers of older adults and what personal capacities carers draw on in managing the systemic work	Community setting. Qualitative study, interviews and focus groups	The caring system is disposed to create disparities, and individual carer capacities are crucial for managing the systemic work required
Roberti et al, 2018^44^	Argentina/UK	Systematic review	To develop understanding of treatment burden experienced by adult patients with CKD and ESKD, with an extended focus on experiences for patients in LMIC	LMIC (community and hospital context). Systematic review	End-stage kidney disease causes high-burden, time-consuming, invasive and exhausting tasks which impact on every aspect of individuals’ lives. Further research into interventions to alleviate treatment burden is needed
Roberti et al, 2021^45^	Argentina	Qualitative study	To describe the status passage that individuals with kidney failure go through to enhance understanding of patient experiences	Hospital (LMIC context). Semi-structured qualitative interviews	Status passage theory illustrated the experiences of patients and the impact of their diagnosis on their lives, with factors such as loss of control, multiplicity, and lack of understanding and information highlighted
Roberti et al, 2022^46^	Argentina	Qualitative study	To illustrate the impact of system control over services, relational networks and social structures on kidney patients’ experiences of their disease and treatment	Hospital setting. Qualitative methods, semi-structured interviews	Patients’ work, experiences and capacity to manage kidney failure is affected by health system control over services, socioeconomic factors and social structures
Tarzia et al, 2016^47^	Australia	Discussion paper	To propose and assess an adapted theoretical framework (based on chronic disease management frameworks) for a web-based domestic violence intervention (I-DECIDE)	Non-clinical community setting. Discussion of case study and proposal of theoretical framework	The modified chronic disease framework provided could strengthen the case for the proposed domestic violence intervention, and displays how the intervention could mobilise capacity and enact positive change in women experiencing domestic violence

Table 2 illustrates that semi-structured interviews, questionnaires or focus groups with patients were carried out in the majority (n= 19) of studies. Five papers used interview methods to explore carers’ experiences,^19,24,33,38,43^ whilst eleven papers sought perspectives of healthcare professionals.^19,24,25,27,29,33–35,37,45,46^ Overall, nine studies sought multiple perspectives (a combination of patients, carers, or healthcare professionals) through interviews, with three studies seeking all three groups’ perspectives.^19,24,33^

Seventeen studies investigated patient experiences of treatment burden across a range of illnesses such as: heart failure,^21,38–40^ kidney disease,^29,38,44–46^ cancer,^26,36,37^ palliative care experiences,^28^ stroke,^34^ COVID-19 effects^23,35^ and contextual multimorbidity experiences.^14^ One study solely explored carer workload.^43^

Ten papers focused on improving service delivery and the implementation of clinical interventions.^19,20,22,24,25,27,31,33,41,42^ One explored improvement for safety in primary care,^33^ whilst another reviewed the value and optimisation of group diabetes clinics for young people,^42^ and four investigated digital healthcare interventions^20,27,31,41^- for example, a digital asthma self-management intervention.^20^ The singular paper with a non-clinical setting also focused on a digital intervention- Tarzia et al discussed the feasibility of a web-based domestic violence intervention^47^. Lastly, a theory discussion paper proposed a middle-range theory to aid in illustrating capacity and accountability.^30^

### Applications of BOTT

As shown in Table 3, most (n= 27) studies prospectively outlined use of BOTT, with just three^29,32,33^ applying BOTT retrospectively to report study outputs^33^ and ‘inform the analysis’ of findings.^29,32^

**Table 3.** The application of BOTT, retrospective or prospective, additional theory use and level of discussion of BOTT constructs of included studies.

First author, year	Country	Application of BOTT	BOTT applied retrospectively or prospectively?	Theories used alongside BOTT	Discussion of BOTT constructs
Abu Dabrh et al, 2021^19^	United States	BOTT was included in the final coding book to underpin data analysis	Prospectively	Cumulative complexity model, theory of patient capacity	No discussion of BOTT constructs, general discussion of workload and capacity centred on pillars of MDM, no further references to BOTT made
Ainsworth et al, 2019^20^	UK	BOTT used to develop questionnaire for assessment of patient burden	Prospectively	None	No discussion of BOTT constructs
Austin et al, 2021^21^	North America, Asia, Europe, Africa	BOTT used as the framework for analysis	Prospectively	None	Defined and discussed BOTT and its key theoretical domains of patient capacity, workload and impact. Explained how BOTT provided a ‘patient-focused framework’ for this review
Brunelli et al, 2021^22^	Australia	BOTT used to frame case study discussion	Prospectively	Cumulative complexity model	Discussed capacity and the importance of relational networks, as well as the concept of variation between individuals’ cognitive and material resources which impair their ability to manage their burden
Callan et al, 2021^23^	UK	BOTT used as a theoretical lens to inform analysis	Prospectively	None	No discussion of BOTT constructs
Chikumbu et al, 2022^14^	Malawi	BOTT used to theoretically inform analysis and to structure the analytical account given, as well as being adapted for and applied to an LMIC context	Prospectively	Normalisation process theory	Constructs and conceptualisation of BOTT described and summarised. Study findings presented and mapped onto BOTT constructs and ‘generative principles’. BOTT model refined and extended to include ‘lack of treatment’. Detailed discussion
Corbett et al, 2020^24^	UK	BOTT used to contextualise methods of intervention development	Prospectively	Cumulative complexity model, cognitive authority theory, self determination theory	A brief outline of BOTT constructs given in table; outlined definition of the theory and its associated aims to justify its use in intervention methods
Early et al, 2018^25^	UK	BOTT used in data analysis to ‘understand patient experiences’	Prospectively	Normalisation process theory, minimally disruptive medicine principles	Definition provided of BOTT as a ‘structural model’ which seeks to illustrate ‘capacity’ of individuals to undertake ‘workload’
Foster et al, 2019^26^	UK	BOTT will be used to underpin data analysis	Prospectively	Conceptual ‘framework’ of recovery of health and wellbeing post-cancer treatment	No discussion of BOTT constructs
Gilbert et al, 2021^27^	UK	BOTT informed design of the interview schedules	Prospectively	None	Briefly defined BOTT in relation to workload and capacity, no further discussion of constructs was provided
Green et al, 2016^28^	UK	BOTT used to guide analysis and ‘extend understanding’	Prospectively	None	Findings mapped to and discussed according to BOTT themes such as ‘capacity for action’, ‘sense making’ and the importance of patient ‘functional performance and social skills’ on capacity mobilisation. Additionally- the importance of ‘social networks’
Hounkpatin et al, 2020^29^	UK	BOTT applied to findings and used to inform analysis	Retrospectively	None	Limited discussion of how findings support BOTT proposal that healthcare engagement is impacted by treatment burden
Hunt et al, 2017^30^	UK	BOTT used to inform theoretical framework development	Prospectively	Normalisation process theory	No discussion of BOTT constructs (language similar to cognitive authority theory)
Husebo et al, 2019^31^	Norway	BOTT used to frame discussion of the presentation of a new theoretical model	Prospectively	None	Limited discussion of BOTT constructs, definition of BOTT provided
Jakukbowski et al, 2022^32^	North America, Asia, Australia, Europe	BOTT used to revise coding framework and then used in data analysis and interpretation	Retrospectively	None	Defined BOTT and how it related to this study, reference to constructs such as ‘sense-making’, support networks and patient work
Knowles et al, 2017^33^	UK	BOTT was used to frame study discussions and outputs- not during analysis	Retrospectively	None	The results are framed with reference to BOTT and include the concepts of ‘capacity’, ‘personal and social context’, ‘sense-making’ work for mobilising action, and relational networks
Kyle et al, 2020^34^	UK	BOTT informed the interview schedule	Prospectively	None	No discussion of BOTT constructs
Ladds et al, 2020^35^	UK	BOTT informed the design of question prompts for the interviews	Prospectively	None	No discussion, referred to BOTT as ‘burden of illness theory’
Lippiett et al, 2018^36^	Europe, north America, Australia	BOTT was used to underpin the coding framework	Prospectively	Status passage theory	Defined BOTT and workload, explained constructs of fluctuating capacity and the importance of social networks
Lippiett et al, 2022^37^	UK	Study ‘built on burden of treatment theory’ to develop a taxonomy of patient experiences with workload	Prospectively	Status passage theory	Discussion and reference to BOTT constructs such as ‘collective illness identities’, ‘structural resilience’ and ‘capacity’
May et al, 2015^38^	UK	BOTT used to inform intervention development	Prospectively	Normalisation process theory	Limited discussion of constructs, refers to ‘relational networks’ and their capacity
Nordfonn et al, 2019^39^	Norway	BOTT used to inform question routes	Prospectively	None	Discussed BOTT framework and its importance for treatment burden and capacity. Discusses the importance of the emotional dimension in burden of treatment generally
Nordfonn et al, 2020^40^	Norway	BOTT informed design of interview questions	Prospectively	None	Discussed the construct of capacity in relation to BOTT, as well as the importance of social skills and social capital on treatment burden management
O’Connor et al, 2016^41^	UK	BOTT used as a ‘lens’ to develop recommendations for engagement strategies for digital health interventions	Prospectively	Normalisation process theory	All recommendations outlined and explained with close reference to BOTT constructs and concepts (sense-making, ‘relational networks, functional performance, social capital)
Papoutsi et al, 2019^42^	UK	BOTT used as a ‘sensitising lens’ for the literature review and research questions	Prospectively	Referenced several chronic disease self-management theories	No discussion of BOTT constructs
Quigley et al, 2021^43^	Australia	BOTT applied as a conceptual approach to inform methods of development	Prospectively	None	Some discussion of BOTT constructs, outlined idea that workload has ‘shifted’ to patient networks and requires coordination
Roberti et al, 2018^44^	Argentina/UK	BOTT concepts used in the coding framework	Prospectively	Cognitive authority theory	Brief outline of theory definition, no further discussion of BOTT constructs
Roberti et al, 2021^45^	Argentina	BOTT was used to inform the coding framework that was applied to a qualitative literature synthesis	Prospectively	Status passage theory, cognitive authority model	No discussion of BOTT constructs
Roberti et al, 2022^46^	Argentina	The theoretical constructs of BOTT informed the qualitative literature synthesis, data collection and data analysis in this study	Prospectively	Status passage theory, cognitive authority model	Defined BOTT and its constructs of ‘sense-making’, ‘cognitive participation’, ‘collective action’ and ‘reflexive monitoring’
Tarzia et al, 2016^47^	Australia	BOTT was adapted and applied to provide a framework for a new theoretical model of a domestic violence intervention	Prospectively	Normalisation process theory	Constructs of BOTT defined- capacity, relational networks and agency as ‘resources to be mobilised’ are outlined, as well as access to ‘social capital’. BOTT framework was adapted to fit the ‘real world’ context and allow analysis of the intervention- the proposed model focuses on capacity for engaging in ‘strategies for safety and wellbeing’ rather than ‘to interact with or utilise healthcare services’

Six studies stated use of BOTT to inform development of interview schedules or questionnaires,^20,27,34,35,39,40^ whilst a further five used BOTT as framework for their final coding books.^19,32,36,44,45^ The provision of a rationale for these applications of BOTT varied, with five studies merely stating use^19,20,34,35,45^ whilst others drew parallels between BOTT design and study aims – for example, Nordfonn et al outlined how the BOTT concept of ‘capacity’ aligned with their aim to explore heart failure patients’ perceptions of treatment burden.^40^

The use of BOTT to underpin or inform data analysis was the stated application in a further eight papers.^14,21,23,25,26,28,29,46^ Level of rationale for BOTT use again varied. Chikumbu et al outlined that BOTT appeared to ‘support a generalizable understanding of multimorbidity’ and aimed to assess its utility for ‘structuring analysis of patient experience’ in a Low-or-Middle-Income Country (LMIC) context.^14^ Other studies justified BOTT use broadly to ‘understand patient experience’,^25^ or ‘extend understanding’ by using an ‘established theory’.^28^

Two studies with a research focus on the effects of COVID-19 incorrectly referred to BOTT as the ‘Burden of Illness Theory’.^23,35^ The stated uses of the theory were to inform data analysis^23^ and to design question prompts,^35^ and neither study further elaborated on theory usage.

Three studies used BOTT to theoretically enhance their approach to methodology,^37,42,43^ with one describing BOTT as a ‘sensitising lens’ for development of their literature review questions,^42^ and another as ‘a conceptual approach’ for their exploration of carer workload experiences.^43^ Bruneli et al used BOTT to frame a case study discussion characterising disability workload during COVID-19.^22^

Seven papers used BOTT to inform or discuss methods of intervention development.^24,30,31,33,38,41,47^ One protocol aimed to build on BOTT development when designing their conceptual model, as they felt BOTT was useful for ‘negotiating and embedding processes of care’,^38^ whilst another used BOTT as ‘a lens to develop recommendations’ for digital health engagement.^41^ Tarzia et al adapted and applied BOTT to assess whether their domestic violence intervention ‘might increase women’s agency and capacity for action’ and proposed a theoretical model using BOTT,^47^ whilst Corbett et al outlined that using the ‘theoretical framework’ of BOTT in their multimorbidity intervention would enable them to ‘ensure relevant factors identified in theory are addressed in the intervention developed’.^24^

### Discussion of BOTT constructs

Nine papers did not further illustrate theory usage beyond their initial statement of application in methodology and did not include any discussion of BOTT constructs.^19,20,23,26,30,34,35,42,45^ All remaining papers (n=21) provided a rationale for BOTT use and demonstrated engagement with the theory by illustrating or defining BOTT principles to some extent. For example, Hounkpatin et al outlined the BOTT principle that treatment burden influences ‘the extent to which patients can engage in healthcare and everyday responsibilities and relationships’,^29^ whilst May et al acknowledged BOTT as illustrating patients’ abilities to ‘take on self-care and healthcare tasks’.^38^

Thirteen papers critically engaged with BOTT in more detail and used its constructs to illustrate outputs or discussions^14,21,22,28,32,33,36,37,39–41,46,47^. The BOTT concept of capacity was discussed by all thirteen studies. Green et al, who utilised BOTT for analysis of the ‘work required to access emergency care’, mapped study results to BOTT themes such as ‘capacity for action’ (ability to interact with healthcare services and resources) to explain how often patients felt that their ‘capacity to participate’ in healthcare management was restricted by their illness.^28^

The BOTT description of ‘mobilisation’ and ‘expression’ of capacity (methods by which individuals actively engage with and use healthcare networks) were discussed in five studies.^14,28,33,37,47^ One study outlined the importance of patient skill when ‘communicating needs’,^33^ whilst another discussed how requirements for patients to ‘manage and communicate information’ can affect their ‘capacity to mobilise’ when using healthcare services.^28^

Nine studies discussed the BOTT principle that when workload exceeds capacity, treatment burden is exacerbated.^14,21,22,28,36,37,39,40,46^ Lippiett et al refer to the imbalance as ‘a primary driver’ of treatment burden,^36^ whilst a case study discussion highlighted that ‘when health-related work exceeds individual and network capacity’, the ability to ‘perform even simple tasks becomes tenuous’.^22^

The BOTT concepts of ‘social skill’ (an individual’s level of engagement and cooperation with others) and ‘social capital’ (extent of an individual’s ability to access resources and information) in relation to patient capacity were highlighted and explained by ten papers.^14,22,28,33,36,37,40,41,46,47^ Chikumbu et al mapped their study findings to BOTT and observed ‘those able to enlist others in their care were able to increase their capacity’ and ‘social connections provided some with access to information’.^14^ O’Connor et al, who mapped recommendations for digital health interventions to BOTT, highlighted the importance of access to ‘encourage engagement’ with digital interventions.^41^ The ‘functional performance’ of patients (their ability to process and make sense of their healthcare) was another highlighted theme of four included papers,^14,28,41,46^ with one study outlining in results that ‘health literacy and cognitive abilities’ were ‘significant mediators of capacity’^14^ and another recommending that design of digital health interventions be ‘tailored to lessen’ the burden for patients’ ‘cognitive and material capacity’.^41^

‘Relational networks’ outlined in BOTT refer to social networks patients interact with that provide support, extending beyond family and friends. Green et al outlined that patients reporting high levels of social support were able to ‘maintain a sense of independence and interaction’ and also highlighted how this support ‘extended’ to include their healthcare professionals in cases of long-term illnesses, referencing this BOTT principle.^28^ A further five papers discussed relational networks and their importance in providing support^28,32,41,46,47^- Tarzia et al, who adapted the theory to assess a domestic violence intervention, outline that a woman experiencing domestic abuse is more likely to have ‘capacity for action’ if she receives ‘good social support’ from ‘family members and friends, health practitioners, or other abused women’ and state that ‘connections within the community’ are ‘critical to a woman’s journey towards positive change’.^47^

### BOTT use with other theories

Most (n=16) of the included studies used other theories alongside BOTT including: Normalisation Process Theory (NPT) (n=6)^14,25,30,38,41,47^, The Cumulative Complexity Model (N=3),^19,22,24^ Cognitive Authority Theory (n=4),^24,44–46^ Status Passage Theory,^36,37^ The Theory of Patient Capacity,^19^ the use of multiple ‘chronic disease self-management theories’^42^ and one outlining use of a conceptual ‘framework’ of recovery.^26^ Fourteen papers did not use any other theory with BOTT.

All six included papers that applied BOTT to develop interview schedules or questionnaires did not use any other theory.^20,27,34,35,39,40^ Four of the five papers that used BOTT for coding frameworks used additional theories, including Cognitive Authoritative Theory, Status Passage Theory and the Cumulative Complexity Model, alongside BOTT in their frameworks.^19,36,44,45^ Whilst papers that used NPT in conjunction with BOTT most often used the theory to inform or develop methods of intervention,^30,38,41,47^ 2 papers that used BOTT to aid in data analysis also outlined NPT use.^14,25^

### BOTT adaptations and extensions

**Figure 3.**
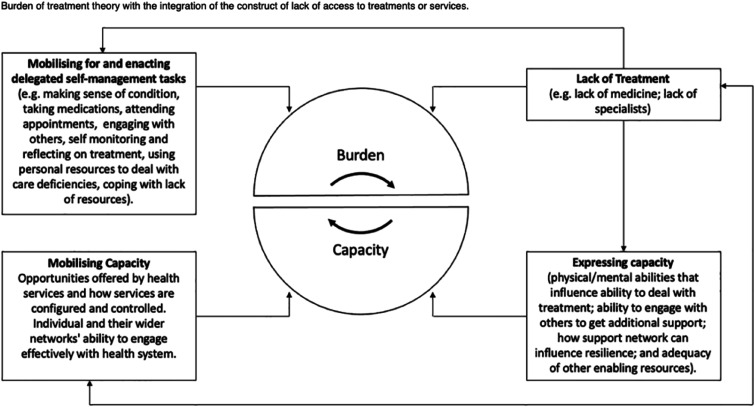
The BOTT framework with the added concept of ‘lack of treatment’ incorporated, from Chikumbu et al.^14^

Two papers adapted or refined BOTT to the context of their research focus- Chikumbi et al, who carried out a study on treatment burden experiences in Malawi, proposed to integrate the concept of ‘lack’ of treatment to BOTT framework (see Figure 3), outlining that lack of access is ‘an important additional dimension’ in LMICs and ‘socioeconomically disadvantaged populations more widely’.^14^

**Figure 4.**
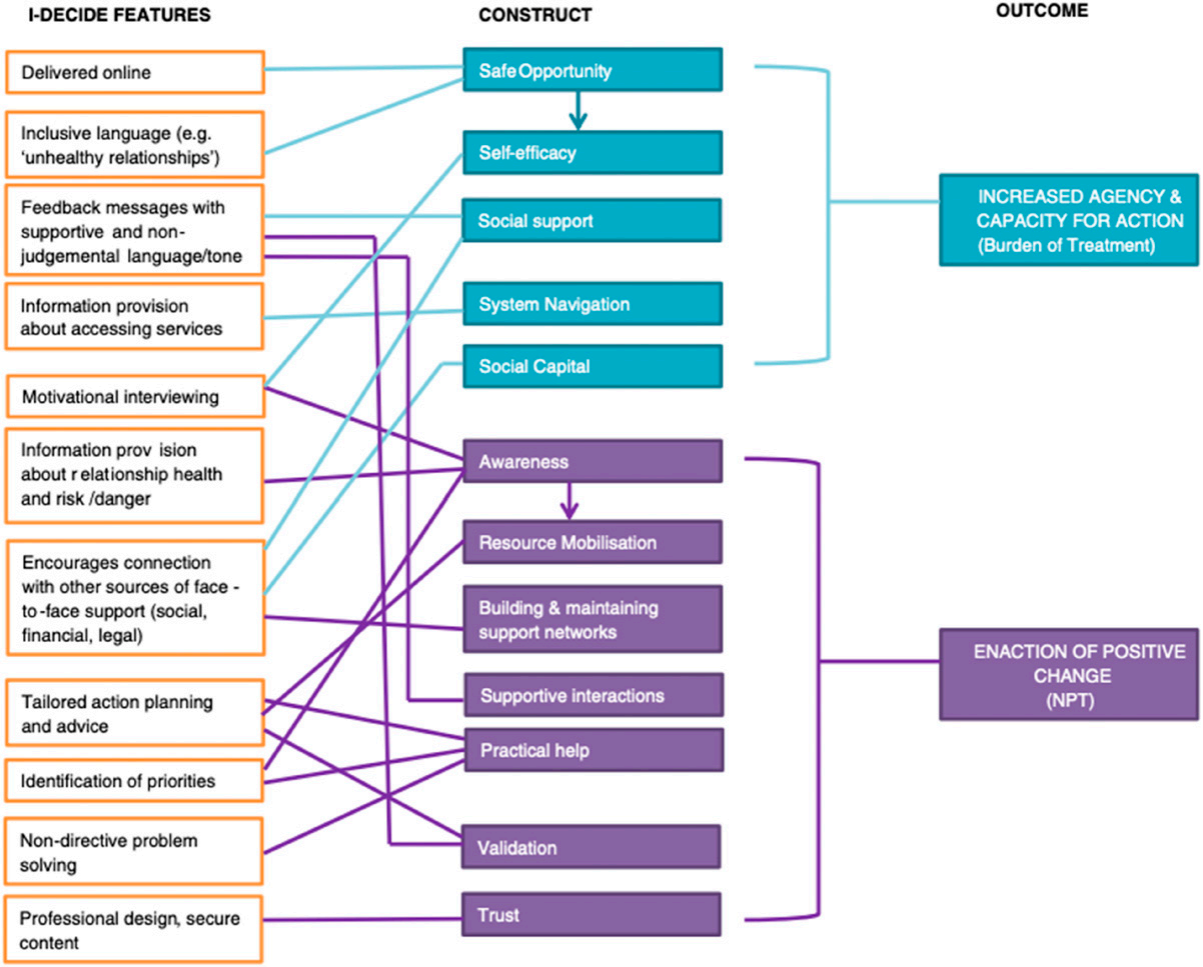
Features of I-DECIDE, the domestic violence intervention, mapped to BOTT and NPT constructs, from Tarzia et al.^47^

In the only paper with a non-clinical setting, Tarzia et al adapted the chronic disease elements of the BOTT framework to make them applicable to victims of intimate partner violence, altering the focus of BOTT from ‘capacity of individuals and their relational networks to interact with and utilise healthcare services’ to ‘capacity of women and their support networks to engage in strategies for safety and wellbeing’.^47^ The features of their proposed model were mapped to the constructs of both BOTT and NPT (see Figure 4).

### BOTT commentary and contribution to findings

BOTT was broadly described, applied and discussed aptly by researchers, and papers that critically engaged with the theory had applied it across a variety of different contexts to contribute to a range of outcomes relevant to improving understanding of treatment burden and capacity issues for self-management.

Four papers commented more specifically on the adaptability and usefulness of BOTT. Nordfonn et al remarked that BOTT contributed to ‘understanding of the challenges of living with heart failure’.^39^ Knowles et al outlined that BOTT constructs fit well with their results, which ‘demonstrate the value of this theory to understanding, and potentially improving, patient safety in primary care’.^33^

Chikumbu et al commented that BOTT highlighted how ‘social networks and the resources which they can bring’ can aid in lessening treatment burden.^14^ Additionally, whilst this study stated that BOTT was suitable to enable ‘conceptualisation of treatment burden issues in LMICs’, they additionally outlined that the perceived missing concept of ‘lack’ of treatment ‘merits further investigation’, and recommended its integration into future measures of treatment burden to redress BOTT’s ‘implicit assumption’ that treatment is ‘available’.^14^

Some studies made general comments about usefulness of theoretical frameworks in qualitative research but did not elaborate further. Roberti et al remarked that the use of framework analysis improved transparency of coding, improving the robustness of their study,^44^ whilst Tarzia et al commented that ‘theories around effective self-management’ proved useful to assess interventions with a ‘real-world setting’.^47^ Corbett et al highlighted that the ‘use of theory’ in methodology allowed ‘a holistic approach to support those with multimorbidity’.^24^

## Discussion

BOTT has been applied in a broad range of settings to support qualitative research design and methodology, as well as to frame discussions. Research focused on the characterisation of treatment burden in specific diseases and contexts, as well as the advancement of interventions to reduce treatment burden. BOTT was most commonly used prospectively for data analysis or to underpin data collection methods, and the second most common application was to inform methods of intervention development. The wide range of healthcare settings across many specialties, including a non-clinical application, demonstrates the flexibility of BOTT and implies good adaptability and usability for issues of treatment burden, self-care and capacity.

Studies included a range of patient, carer and health professional experiences, which accurately reflects the intended multi-perspective themes of BOTT.

Analysis of the application of BOTT showed that most prospective explanations for theoretical usage aligned with the intended purpose of the theory, revealing that general understanding and stability of the constructs across different settings is high. However, discussion and mapping of results and interventions to BOTT constructs was less widespread, as were adaptations of BOTT. This indicates the theory was well-suited to these study contexts, as most studies originated in high-income settings similar to the development setting of BOTT.

The papers which adapted BOTT framework both were in contexts different to that in which BOTT was developed- with one study adapting BOTT to a non-healthcare setting,^46^ and another extending BOTT to better suit an LMIC setting, as they proposed that ‘lack’ of treatment was a missing construct.^14^ This is a valuable reflection for future iterations of the theory in order to increase its generalisability further. However, as a middle-range theory, BOTT use should be both flexible and critical^48^ and the fact that these adaptations have been made successfully indicates that researchers are able to engage critically with the theory appropriately.

Reflection on the conceptual toolkit provided by the theory and its contribution to findings was rare, although several authors did acknowledge the importance of theoretically informed research.^24,44,47^ When provided, commentary on the theory was positive about its value and contribution to findings.^14,32,33,39^ Interestingly, most studies used other theories in conjunction with BOTT to inform research, such as NPT. The use of multiple theories in coding frameworks or data collection can be useful for illustrating multiple facets,^49^ and more established theories in the literature like NPT are perhaps more widely understood compared with the relatively recent BOTT.

Most studies listed an author of the original BOTT conceptual paper, perhaps due to its recent development and the specialised field of modern treatment burden research. However, in order to further ascertain how the constructs are understood and if current published literature is adequate for explaining application, it would be useful to assess independent research teams’ applications of BOTT.

### Strengths and limitations

The wide variety of methodologies and applications made synthesis and interpretation of BOTT application complex. However, the heterogeneity of papers allowed comprehensive characterisation of BOTT use, which increases the validity of our findings. Systematic reviews were included in this review to allow a comprehensive examination of all research that has utilised BOTT, including use of the theory in research synthesis. The original research studies in the included systematic reviews were not included in our review unless they had utilised BOTT in their methods. Although typically systematic reviews are not included in traditional systematic reviews of interventional studies, there are precedents for this in systematic reviews of theory.^15^ The inclusion of four LMIC-focused studies extends our understanding of the theory and outlines that BOTT is transferable- although due to the low number of these studies included, we cannot extrapolate further the efficacy of the theory in these contexts. The exclusion of non-English language papers may have restricted illustrating theory use geographically from areas of more socioeconomic deprivation.

To improve the transparency and reliability of our review, all steps were led by RS who had no prior involvement in theory development. Our search strategy focused on finding papers that had cited and engaged with BOTT in keeping with our research question, rather than the use of key terms as would typically be used in systematic reviews of interventional studies. Second and third reviewers for the processes of screening, extraction and analysis added to the robustness and validity of findings. All papers were double reviewed at these steps as is recommended by Cochrane guidance.^50^ Reproducibility of the data screening and extraction process is high due to the detailed data extraction form developed and the use of DistillerSR software, increasing audibility and validity of the research.^51^ However, there is a level of researcher subjectivity that is unavoidable in qualitative data analysis,^52^ and so different researchers may reach slightly different conclusions on theory utility or commentary, as informed by their personal background of BOTT. Regular discussion around extraction process steps and thematic constructs of BOTT with experts in the field ensured focus and interpretation was agreed upon and understood.

### Contribution to treatment burden research

Treatment burden research has proliferated since the proposal of Minimally Disruptive Medicine in 2009, a clinical strategy which outlined the need for treatment services that minimised user workload.^1^ Theoretical frameworks addressing patient workload,^6–8^ capacity^9^ and the characterisation of the relationships between them,^11^ and the production of patient-reported measures of treatment burden informed by these theories and frameworks^10,53,54^ have all contributed to make illness management less disruptive for patients and their support networks.^55^ BOTT is one of the more recent theories to join this body of literature, and this systematic review demonstrates the contribution and validity of its use in research seeking to both illustrate and alleviate treatment burden. NPT, another theory which advocates for minimally disruptive medicine in practice, has similarly undergone review to assess theoretical application and contribution.^15,16^ These reviews informed application and were able to direct future NPT literature to maximise theoretical contribution. By reviewing BOTT in a similar manner, this review illustrates the use and contribution of the theory both to healthcare interventions and to research, and similarly provides recommendations to direct future BOTT work.

### Recommendations and implications for future research

Whilst the prospective use of BOTT to inform research methods and provide a conceptual lens for analysis is a positive finding of this review, BOTT has the potential to be further utilised adaptively in a range of settings and cultures. There are many clinical settings not yet explored, for example it would be interesting to explore the nuanced work that relates to different clusters of conditions. The burden of treatment experience by unpaid carers of people with long-term conditions is under-researched and should be given priority. It is also important to explore burden of treatment in settings other than the high-income country in which BOTT was developed.^14^ Future research could explore and discuss the contextual relevance of BOTT to specific cultural and socioeconomic settings in order to facilitate continued refinement and adaptability of BOTT. Over time as more independent researcher groups with no connection to theory creation increasingly utilise and critically engage with this theory, further reviews may be able to ascertain a broader picture of the scope of its usability, as was found in a second systematic review of NPT.^16^

## Conclusion

BOTT provides a useful conceptual, analytical and sensitising lens in studies focusing on both the characterisation and alleviation of treatment burden in those with chronic illness and multimorbidity through healthcare interventions. The constructs discussed are stable and applicable to a wide range of settings. Future BOTT literature could include its utilisation by empirical researchers in contexts which would require more adaptation and critical assessment of the theory.

## Supplemental Material

Supplemental Material - A systematic review of the use of burden of treatment theory Supplemental Material for A systematic review of the use of burden of treatment theory by Rachel C Smyth, Georgia Smith, Emily Alexander, Carl R May, Frances S Mair, and Katie I Gallacher in Journal of Multimorbidity and Comorbidity

Supplemental Material - A systematic review of the use of burden of treatment theory Supplemental Material for A systematic review of the use of burden of treatment theory by Rachel C Smyth, Georgia Smith, Emily Alexander, Carl R May, Frances S Mair, and Katie I Gallacher in Journal of Multimorbidity and Comorbidity

Supplemental Material - A systematic review of the use of burden of treatment theory Supplemental Material for A systematic review of the use of burden of treatment theory by Rachel C Smyth, Georgia Smith, Emily Alexander, Carl R May, Frances S Mair, and Katie I Gallacher in Journal of Multimorbidity and Comorbidity

Supplemental Material - A systematic review of the use of burden of treatment theory Supplemental Material for A systematic review of the use of burden of treatment theory by Rachel C Smyth, Georgia Smith, Emily Alexander, Carl R May, Frances S Mair, and Katie I Gallacher in Journal of Multimorbidity and Comorbidity

Supplemental Material - A systematic review of the use of burden of treatment theory Supplemental Material for A systematic review of the use of burden of treatment theory by Rachel C Smyth, Georgia Smith, Emily Alexander, Carl R May, Frances S Mair, and Katie I Gallacher in Journal of Multimorbidity and Comorbidity
