# Supplementary material for: A systematic review of the use of burden of treatment theory
Source: J Multimorb Comorb. 2025 May 9;15:26335565251314828. doi: 10.1177/26335565251314828 (PMC12064904; doi:10.1177/26335565251314828)
Supplement: Supplemental Material - A systematic review of the use of burden of treatment theory [file sj-pdf-1-cob-10.1177_26335565251314828.pdf]

Distiller SR data download: Exclusions for both Level 1 (Title/Abstract screening) and Level 2 (Full text screening)

Result #	Reference	Exclusion Criteria
175	RefID: 175, Positioning the six-month review in the recovery process post-stroke: The ideology of personal responsibility<br>Abrahamson, V.,Wilson, P.<br>Level: 2, State: Excluded	Level 2, Form level_2_full_text_screening, This study used the Burden of Treatment Theory: -> None of the above
287	RefID: 287, Minimally disruptive medicine: The evidence and conceptual progress supporting a new era of healthcare<br>Abu Dabrh, A. M.,Gallacher, K.,Boehmer, K. R.,Hargraves, I. G.,Mair, F. S.<br>Level: 2, State: Excluded	Level 2, Form level_2_full_text_screening, This study used the Burden of Treatment Theory: -> None of the above
241	RefID: 241, The aesthetic experience of dying: The dance to death<br>Adamson, V. M. F.<br>Level: 1, State: Excluded	Level 1, Form level_1__titleabstract_screeni
516	RefID: 516, What are the core predictors of 'hassles' among patients with multimorbidity in primary care? A cross sectional study<br>Adeniji, Charles,Kenning, Cassandra,Coventry, Peter A.,Bower, Peter<br>Level: 2, State: Excluded	Level 2, Form level_2_full_text_screening, This study used the Burden of Treatment Theory: -> None of the above
139	RefID: 139, Institutionalising emergent organisation in health and social care<br>Allen, D.<br>Level: 1, State: Excluded	Level 1, Form level_1__titleabstract_screeni
267	RefID: 267, Towards a sociology of healthcare safety and quality<br>Allen, D.,Braithwaite, J.,Sandall, J.,Waring, J.<br>Level: 1, State: Excluded	Level 1, Form level_1__titleabstract_screeni
81	RefID: 81, Leadership for careful and kind care<br>Allwood, D.,Koka, S.,Armbruster, R.,Montori, V.<br>Level: 1, State: Excluded	Level 1, Form level_1__titleabstract_screeni
592	RefID: 592, Care Coordination: A Concept Analysis<br>Anderson, Amanda,Mpa, M. S. N.,Hewner, Sharon,PhD, R. N.<br>Level: 2, State: Excluded	Level 2, Form level_2_full_text_screening, This study used the Burden of Treatment Theory: -> None of the above
198	RefID: 198, Being in safe hands: Patients' perceptions of how cancer services may support psychological well-being<br>Appleton, L.,Poole, H.,Wall, C.<br>Level: 2, State: Excluded	Level 2, Form level_2_full_text_screening, This study used the Burden of Treatment Theory: -> None of the above

113	RefID: 113, Managing Diabetes in Hard to Reach Populations: A Review of Telehealth Interventions<br>Appuswamy, A. V.,Desimone, M. E.<br>Level: 1, State: Excluded	Level 1, Form level_1__titleabstract_screeni
614	RefID: 614, SYMptoms in chronic heart failure imPACT on burden of treatment (SYMPACT): a cross-sectional survey<br>Austin, R. C.,Schoonhoven, L.,Koutra, V.,Richardson, A.,Kalra, P. R.,May, C. R.<br>Level: 1, State: Excluded	Level 1, Form level_1__titleabstract_screeni
91	RefID: 91, How do SYMPtoms and management tasks in chronic heart failure imPACT a person's life (SYMPACT)? Protocol for a mixed-methods study<br>Austin, R. C.,Schoonhoven, L.,Richardson, A.,Kalra, P. R.,May, C. R.<br>Level: 2, State: Excluded	Level 2, Form level_2_full_text_screening, This study used the Burden of Treatment Theory: -> None of the above
530	RefID: 530, Burden of treatment in chronic heart failure: does symptom burden play a role?<br>Austin, Rosalynn C.,Schoonhoven, Lisette,Kalra, Paul R.,May, Carl R.<br>Level: 2, State: Excluded	Level 2, Form level_2_full_text_screening, This study used the Burden of Treatment Theory: -> None of the above
429	RefID: 429, Experience and Context Shape Patient and Clinician Goals For Treatment of Rheumatoid Arthritis: A Qualitative Study<br>Barton, Jennifer L.,Hulen, Elizabeth,Schue, Allison,Yelin, Edward H.,Ono, Sarah S.,Tuepker, Anais,Koenig, Christopher J.<br>Level: 2, State: Excluded	Level 2, Form level_2_full_text_screening, This study used the Burden of Treatment Theory: -> None of the above
71	RefID: 71, 2017 National Standards for Diabetes Self-Management Education and Support<br>Beck, J.,Greenwood, D. A.,Blanton, L.,Bollinger, S. T.,Butcher, M. K.,Condon, J. E.,Cypress, M.,Faulkner, P.,Fischl, A. H.,Francis, T.,Kolb, L. E.,Lavin-Tompkins, J. M.,MacLeod, J.,Maryniuk, M.,Mensing, C.,Orzeck, E. A.,Pope, D. D.,Pulizzi, J. L.,Reed, A. A.,Rhinehart, A. S.,Siminerio, L.,Wang, J.<br>Level: 1, State: Excluded	Level 1, Form level_1__titleabstract_screeni
128	RefID: 128, 2017 National Standards for Diabetes Self-Management Education and Support<br>Beck, J.,Greenwood, D. A.,Blanton,	Level 1, Form level_1__titleabstract_screeni

	L.,Bollinger, S. T.,Butcher, M. K.,Condon, J. E.,Cypress, M.,Faulkner, P.,Fischl, A. H.,Francis, T.,Kolb, L. E.,Lavin-Tompkins, J. M.,MacLeod, J.,Maryniuk, M.,Mensing, C.,Orzeck, E. A.,Pope, D. D.,Pulizzi, J. L.,Reed, A. A.,Rhinehart, A. S.,Siminerio, L.,Wang, J.<br>Level: 1, State: Excluded	
165	RefID: 165, 2017 National Standards for Diabetes Self-Management Education and Support<br>Beck, J.,Greenwood, D. A.,Blanton, L.,Bollinger, S. T.,Butcher, M. K.,Condon, J. E.,Cypress, M.,Faulkner, P.,Fischl, A. H.,Francis, T.,Kolb, L. E.,Lavin-Tompkins, J. M.,MacLeod, J.,Maryniuk, M.,Mensing, C.,Orzeck, E. A.,Pope, D. D.,Pulizzi, J. L.,Reed, A. A.,Rhinehart, A. S.,Siminerio, L.,Wang, J.<br>Level: 1, State: Excluded	Level 1, Form level_1__titleabstract_screeni
464	RefID: 464, 2017 National Standards for Diabetes Self-Management Education and Support<br>Beck, Joni,Greenwood, Deborah A.,Blanton, Lori,Bollinger, Sandra T.,Butcher, Marcene K.,Condon, Jo Ellen,Cypress, Marjorie,Faulkner, Priscilla,Fischl, Amy Hess,Francis, Theresa,Kolb, Leslie E.,Lavin-Tompkins, Jodi M.,MacLeod, Janice,Maryniuk, Melinda,Mensing, Carole,Orzeck, Eric A.,Pope, David D.,Pulizzi, Jodi L.,Reed, Ardis A.,Rhinehart, Andrew S.,Siminerio, Linda,Wang, Jing,Stand Revision Task, Force<br>Level: 1, State: Excluded	Level 1, Form level_1__titleabstract_screeni
453	RefID: 453, 2017 National Standards for Diabetes Self-Management Education and Support<br>Beck, Joni,Greenwood, Deborah A.,Blanton, Lori,Bollinger, Sandra T.,Butcher, Marcene K.,Condon, Jo Ellen,Cypress, Marjorie,Faulkner, Priscilla,Fischl, Amy Hess,Francis, Theresa,Kolb, Leslie E.,Lavin-Tompkins, Jodi M.,MacLeod, Janice,Maryniuk, Melinda,Mensing, Carole,Orzeck, Eric A.,Pope, David D.,Pulizzi, Jodi L.,Reed, Ardis A.,Rhinehart, Andrew S.,Siminerio, Linda,Wang, Jing,Stand Revision Task, Force	Level 1, Form level_1__titleabstract_screeni

	Level: 1, State: Excluded	
573	RefID: 573, 2017 National Standards for Diabetes Self-Management Education and Support<br>Beck, Joni,Greenwood, Deborah,PhD, R. N.,Bc-Adm, C. D. E.,Blanton, Lori,Ms, Ches,Bollinger, Sandra,PharmD, C. G. P.,Cde, Fascp,Butcher, Marcene,Rd, C. D. E.,Condon, Jo,Ellen Rdn, C. D. E.,Cypress, Marjorie,PhD, C. Anp,Faulkner, Priscilla,Ms, M. A.,Cns, R. N.,Fischl, Amy,Hess Ms, R. D. N.,Ldn, Bc-Adm,Francis, Theresa,Msn, R. N.,Kolb, Leslie,Mba, B. S. N.,Lavin-Tompkins, Jodi,Msn, R. N.,Bc-Adm, C. D. E.,MacLeod, Janice,Ma, R. D.,Ld, C. D. E.,Maryniuk, Melinda,Med, R. D.,Mensing, Carole,Ma, R. N.,Cde, Faade,Orzeck, Eric,Md, Facp,Face, C. D. E.,Pope, David,PharmD, C. D. E.,Pulizzi, Jodi,Rn, C. D. E.,Reed, Ardis,Mph, R. D.,Ld, C. D. E.,Rhinehart, Andrew,Md, Bc-Adm,Cde, Facp,Siminerio, Linda,PhD, R. N.,Wang, Jing,PhD, M. P. H.<br>Level: 1, State: Excluded	Level 1, Form level_1__titleabstract_screeni
274	RefID: 274, What to expect from the evolving field of geriatric cardiology<br>Bell, S. P.,Orr, N. M.,Dodson, J. A.,Rich, M. W.,Wenger, N. K.,Blum, K.,Harold, J. G.,Tinetti, M. E.,Maurer, M. S.,Forman, D. E.<br>Level: 2, State: Excluded	Level 2, Form level_2_full_text_screening, This study used the Burden of Treatment Theory: -> None of the above
252	RefID: 252, Patient activation in older people with long-term conditions and multimorbidity: Correlates and change in a cohort study in the United Kingdom<br>Blakemore, A.,Hann, M.,Howells, K.,Panagioti, M.,Sidaway, M.,Reeves, D.,Bower, P.<br>Level: 2, State: Excluded	Level 2, Form level_2_full_text_screening, This study used the Burden of Treatment Theory: -> None of the above
212	RefID: 212, Does the chronic care model meet the emerging needs of people living with multimorbidity? A systematic review and thematic synthesis<br>Boehmer, K. R.,Dabrh, A. M. A.,Gionfriddo, M. R.,Erwin, P.,Montori, V. M.<br>Level: 1, State: Excluded	Level 1, Form level_1__titleabstract_screeni
146	RefID: 146, Changing conversations in primary care for patients living with chronic conditions: Pilot and feasibility study of the ICAN Discussion Aid	Level 2, Form level_2_full_text_screening, This study used the Burden of Treatment Theory: -> None of the above

	Boehmer, K. R.,Dobler, C. C.,Thota, A.,Branda, M.,Giblon, R.,Behnken, E.,Organick, P.,Allen, S. V.,Shaw, K.,Montori, V. M.<br>Level: 2, State: Excluded	
609	RefID: 609, Minimally Disruptive Medicine: Progress 10 Years Later<br>Boehmer, K. R.,Gallacher, K. I.,Lippiett, K. A.,Mair, F. S.,May, C. R.,Montori, V. M.<br>Level: 2, State: Excluded	Level 2, Form level_2_full_text_screening, This study used the Burden of Treatment Theory: -> None of the above
258	RefID: 258, Patient capacity and constraints in the experience of chronic disease: A qualitative systematic review and thematic synthesis<br>Boehmer, K. R.,Gionfriddo, M. R.,Rodriguez-Gutierrez, R.,Dabrh, A. M. A.,Leppin, A. L.,Hargraves, I.,May, C. R.,Shippee, N. D.,Castaneda-Guarderas, A.,Palacios, C. Z.,Bora, P.,Erwin, P.,Montori, V. M.<br>Level: 2, State: Excluded	Level 2, Form level_2_full_text_screening, This study used the Burden of Treatment Theory: -> None of the above
164	RefID: 164, Capacity Coaching: A New Strategy for Coaching Patients Living With Multimorbidity and Organizing Their Care<br>Boehmer, K. R.,Guerton, N. M.,Soyring, J.,Hargraves, I.,Dick, S.,Montori, V. M.<br>Level: 2, State: Excluded	Level 2, Form level_2_full_text_screening, This study used the Burden of Treatment Theory: -> None of the above
255	RefID: 255, Meaningful conversations in living with and treating chronic conditions: Development of the ICAN discussion aid<br>Boehmer, K. R.,Hargraves, I. G.,Allen, S. V.,Matthews, M. R.,Maher, C.,Montori, V. M.<br>Level: 2, State: Excluded	Level 2, Form level_2_full_text_screening, This study used the Burden of Treatment Theory: -> None of the above
187	RefID: 187, Patient capacity for self-care in the medical record of patients with chronic conditions: A mixed-methods retrospective study 11 Medical and Health Sciences 1117 Public Health and Health Services<br>Boehmer, K. R.,Kyriacou, M.,Behnken, E.,Branda, M.,Montori, V. M.<br>Level: 2, State: Excluded	Level 2, Form level_2_full_text_screening, This study used the Burden of Treatment Theory: -> None of the above
263	RefID: 263, Pursuing minimally disruptive medicine: Disruption from illness and health care-related demands is correlated with patient capacity<br>Boehmer, K. R.,Shippee, N. D.,Beebe, T. J.,Montori, V. M.<br>Level: 2, State: Excluded	Level 2, Form level_2_full_text_screening, This study used the Burden of Treatment Theory: -> None of the above

253	RefID: 253, Resilient health care: Reconciling work-as-imagined and work-as-done<br>Braithwaite, J.,Wears, R. L.,Hollnagel, E.<br>Level: 1, State: Excluded	Level 1, Form level_1__titleabstract_screeni
147	RefID: 147, ‘Homemade’: Building, mending, and coordinating a care network<br>Bruni, A.,Miele, F.,Piras, E. M.<br>Level: 2, State: Excluded	Level 2, Form level_2_full_text_screening, This study used the Burden of Treatment Theory: -> None of the above
603	RefID: 603, What does the literature mean by social prescribing? A critical review using discourse analysis<br>Calderón-Larrañaga, S.,Greenhalgh, T.,Finer, S.,Clinch, M.<br>Level: 2, State: Excluded	Level 2, Form level_2_full_text_screening, This study used the Burden of Treatment Theory: -> None of the above
132	RefID: 132, Humanization of care and culture of welcome. The bambino gesù children hospital: A case of study<br>Casavecchia, A.<br>Level: 2, State: Excluded	Level 2, Form level_2_full_text_screening, This study used the Burden of Treatment Theory: -> None of the above
161	RefID: 161, Living with and beyond cancer with comorbid illness: a qualitative systematic review and evidence synthesis<br>Cavers, D.,Habets, L.,Cunningham-Burley, S.,Watson, E.,Banks, E.,Campbell, C.<br>Level: 2, State: Excluded	Level 2, Form level_2_full_text_screening, This study used the Burden of Treatment Theory: -> None of the above
143	RefID: 143, Cultural adaptation and psychometric properties of the Chinese Burden of Treatment Questionnaire (C-TBQ) in primary care patients with multi-morbidity<br>Chin, W. Y.,Wong, C. K. H.,Ng, C. C. W.,Choi, E. P. H.,Lam, C. L. K.<br>Level: 1, State: Excluded	Level 1, Form level_1__titleabstract_screeni
608	RefID: 608, Experiences of People with Cancer from Rural and Remote Areas of Western Australia Using Supported Accommodation in Perth While Undergoing Treatment<br>Chua, A.,Nguyen, E.,Puah, L. L.,Soong, J.,Keesing, S.<br>Level: 2, State: Excluded	Level 2, Form level_2_full_text_screening, This study used the Burden of Treatment Theory: -> None of the above
601	RefID: 601, Time Spent Engaging in Health Care Among Patients With Left Ventricular Assist Devices<br>Chuzi, S.,Ahmad, F. S.,Wu, T.,Argaw, S.,Harap, R.,Grady, K. L.,Rich, J. D.,Pham, D. T.,Khan, S. S.,Wilcox, J. E.,Allen, L. A.,Tibrewala, A.	Level 2, Form level_2_full_text_screening, This study used the Burden of Treatment Theory: -> None of the above

	Level: 2, State: Excluded	
178	RefID: 178, Hospitalization and post-discharge care in South Africa: A critical event in the continuum of care<br>Cichowitz, C.,Pellegrino, R.,Motlhaoleng, K.,Martinson, N. A.,Variava, E.,Hoffmann, C. J.<br>Level: 2, State: Excluded	Level 2, Form level_2_full_text_screening, This study used the Burden of Treatment Theory: -> None of the above
600	RefID: 600, Self-management by older people living with cancer and multi-morbidity: a qualitative study<br>Corbett, T.,Lee, K.,Cummings, A.,Calman, L.,Farrington, N.,Lewis, L.,Young, A.,Richardson, A.,Foster, C.,Bridges, J.<br>Level: 2, State: Excluded	Level 2, Form level_2_full_text_screening, This study used the Burden of Treatment Theory: -> None of the above
218	RefID: 218, Systematic review of smartphone-based passive sensing for health and wellbeing<br>Cornet, V. P.,Holden, R. J.<br>Level: 1, State: Excluded	Level 1, Form level_1__titleabstract_screeni
243	RefID: 243, Cancer survivors' experience with telehealth: A systematic review and thematic synthesis<br>Cox, A.,Lucas, G.,Marcu, A.,Piano, M.,Grosvenor, W.,Mold, F.,Maguire, R.,Ream, E.<br>Level: 2, State: Excluded	Level 2, Form level_2_full_text_screening, This study used the Burden of Treatment Theory: -> None of the above
68	RefID: 68, Integrating clinical pharmacists within general practice: Protocol for a pilot cluster randomised controlled trial<br>Croke, A.,Moriarty, F.,Boland, F.,McCullagh, L.,Cardwell, K.,Smith, S. M.,Clyne, B.<br>Level: 2, State: Excluded	Level 2, Form level_2_full_text_screening, This study used the Burden of Treatment Theory: -> None of the above
185	RefID: 185, Spatial Associations of Multiple Chronic Conditions Among Older Adults<br>Cromley, E. K.,Wilson-Genderson, M.,Heid, A. R.,Pruchno, R. A.<br>Level: 2, State: Excluded	Level 2, Form level_2_full_text_screening, This study used the Burden of Treatment Theory: -> None of the above
110	RefID: 110, Overruling uncertainty about preventative medications: the social organisation of healthcare professionals' knowledge and practices<br>Cupit, C.,Rankin, J.,Armstrong, N.,Martin, G. P.<br>Level: 2, State: Excluded	Level 2, Form level_2_full_text_screening, This study used the Burden of Treatment Theory: -> None of the above
189	RefID: 189, Safety work and risk management as burdens of treatment in primary care: Insights from a focused ethnographic study of	Level 2, Form level_2_full_text_screening, This study used the Burden of Treatment

	patients with multimorbidity<br>Daker-White, G.,Hays, R.,Blakeman, T.,Croke, S.,Brown, B.,Esmail, A.,Bower, P.<br>Level: 2, State: Excluded	Theory: -> None of the above
109	RefID: 109, Training in health coaching skills for health professionals who work with people with progressive neurological conditions: A realist evaluation<br>Davies, F.,Wood, F.,Bullock, A.,Wallace, C.,Edwards, A.<br>Level: 2, State: Excluded	Level 2, Form level_2_full_text_screening, This study used the Burden of Treatment Theory: -> None of the above
611	RefID: 611, 2022 National Standards for Diabetes Self-Management Education and Support<br>Davis, J.,Fischl, A. H.,Beck, J.,Browning, L.,Carter, A.,Condon, J. E.,Dennison, M.,Francis, T.,Hughes, P. J.,Jaime, S.,Lau, K. H. K.,McArthur, T.,Karen, M.,Magee, M.,Newby, O.,Ponder, S. W.,Quraishi, U.,Rawlings, K.,Socke, J.,Stancil, M.,Uelmen, S.,Villalobos, S.<br>Level: 1, State: Excluded	Level 1, Form level_1__titleabstract_screeni
610	RefID: 610, 2022 National Standards for Diabetes Self-Management Education and Support<br>Davis, J.,Fischl, A. H.,Beck, J.,Browning, L.,Carter, A.,Condon, J. E.,Dennison, M.,Francis, T.,Hughes, P. J.,Jaime, S.,Lau, K. H. K.,McArthur, T.,McAvoy, K.,Magee, M.,Newby, O.,Ponder, S. W.,Quraishi, U.,Rawlings, K.,Socke, J.,Stancil, M.,Uelmen, S.,Villalobos, S.<br>Level: 1, State: Excluded	Level 1, Form level_1__titleabstract_screeni
157	RefID: 157, Assessment of functional health literacy in Brazilian carers of older people<br>de Almeida, K. M. V.,Toye, C.,Silveira, L. V. A.,Slatyer, S.,Hill, K.,Jacinto, A. F.<br>Level: 1, State: Excluded	Level 1, Form level_1__titleabstract_screeni
83	RefID: 83, 'Like a fish on dry land': an explorative qualitative study into severe asthma and the impact of biologicals on patients' everyday life<br>de Graaff, M. B.,Bendien, S. A.,van de Bovenkamp, H. M.<br>Level: 2, State: Excluded	Level 2, Form level_2_full_text_screening, This study used the Burden of Treatment Theory: -> None of the above
619	RefID: 619, 'Like a fish on dry land': an explorative qualitative study into severe asthma and the impact of biologicals on	Level 2, Form level_2_full_text_screening, This study used the Burden of Treatment

	patients’ everyday life<br>de Graaff, M. B.,Bendien, S. A.,van de Bovenkamp, H. M.<br>Level: 2, State: Excluded	Theory: -> None of the above
281	RefID: 281, Living with, managing and minimising treatment burden in long term conditions: A systematic review of qualitative research<br>Demain, S.,Gonçalves, A. C.,Areia, C.,Oliveira, R.,Marcos, A. J.,Marques, A.,Parmar, R.,Hunt, K.<br>Level: 2, State: Excluded	Level 2, Form level_2_full_text_screening, This study used the Burden of Treatment Theory: -> None of the above
115	RefID: 115, The role of virtual reality in improving health outcomes for community-dwelling older adults: Systematic review<br>Dermody, G.,Whitehead, L.,Wilson, G.,Glass, C.<br>Level: 1, State: Excluded	Level 1, Form level_1__titleabstract_screeni
205	RefID: 205, Development and validation of the Multimorbidity Treatment Burden Questionnaire (MTBQ)<br>Duncan, P.,Murphy, M.,Man, M. S.,Chaplin, K.,Gaunt, D.,Salisbury, C.<br>Level: 1, State: Excluded	Level 1, Form level_1__titleabstract_screeni
191	RefID: 191, Palliative and end of life care of people with diabetes: Issues, challenges and strategies<br>Dunning, T.,Martin, P.<br>Level: 2, State: Excluded	Level 2, Form level_2_full_text_screening, This study used the Burden of Treatment Theory: -> None of the above
383	RefID: 383, Pulmonary rehabilitation referral and uptake from primary care for people living with COPD: a mixed-methods study<br>Early, Frances,Wilson, Patricia Mary,Deaton, Christi,Wellwood, Ian,Haque, Hena Wali,Fox, Sarah Emma,Yousaf, Azka,Meysner, Oliver D.,Ward, James R.,Singh, Sally J.,Fuld, Jonathan Paul<br>Level: 2, State: Excluded	Level 2, Form level_2_full_text_screening, This study used the Burden of Treatment Theory: -> None of the above
219	RefID: 219, Making sense of self-care practices at the intersection of severe mental illness and physical health—An Australian study<br>Ehrlich, C.,Chester, P.,Kisely, S.,Crompton, D.,Kendall, E.<br>Level: 2, State: Excluded	Level 2, Form level_2_full_text_screening, This study used the Burden of Treatment Theory: -> None of the above
226	RefID: 226, Unmet expectations of medications and care providers among patients	Level 2, Form level_2_full_text_screening, This

	with heart failure assessed to be poorly adherent: Results from the Chronic Heart Failure Intervention to Improve MEdication Adherence (CHIME) study<br>Ekman, I.,Wolf, A.,Vaughan Dickson, V.,Bosworth, H. B.,Granger, B. B.<br>Level: 2, State: Excluded	study used the Burden of Treatment Theory: -> None of the above
615	RefID: 615, Design and development of an ehealth service for collaborative self-management among older adults with chronic diseases: A theory-driven user-centered approach<br>Ekstedt, M.,Kirsebom, M.,Lindqvist, G.,Kneck,<br>Level: 2, State: Excluded	Level 2, Form level_2_full_text_screening, This study used the Burden of Treatment Theory: -> None of the above
328	RefID: 328, Treatment burden experienced by patients with lung cancer<br>El-Turk, Nicole,Chou, Michael S. H.,Ting, Natasha C. H.,Girgis, Afaf,Vinod, Shalini K.,Bray, Victoria,Dobler, Claudia C.<br>Level: 2, State: Excluded	Level 2, Form level_2_full_text_screening, This study used the Burden of Treatment Theory: -> None of the above
276	RefID: 276, Trustworthy guidelines - excellent; customized care tools - even better<br>Elwyn, G.,Quinlan, C.,Mulley, A.,Agoritsas, T.,Vandvik, P. O.,Guyatt, G.<br>Level: 1, State: Excluded	Level 1, Form level_1__titleabstract_screeni
585	RefID: 585, Clinical encounters in the post-guidelines era<br>Elwyn, Glyn,Wieringa, Siestse,Greenhalgh, Trisha<br>Level: 1, State: Excluded	Level 1, Form level_1__titleabstract_screeni
342	RefID: 342, The burden of treatment in people living with type 2 diabetes: A qualitative study of patients and their primary care clinicians<br>Espinoza, Pilar,Varela, Camila A.,Vargas, Ivonne E.,Ortega, Galo,Silva, Paulo A.,Boehmer, Kasey B.,Montori, Victor M.<br>Level: 2, State: Excluded	Level 2, Form level_2_full_text_screening, This study used the Burden of Treatment Theory: -> None of the above
97	RefID: 97, Known-groups validity and responsiveness to change of the Patient Experience with Treatment and Self-management (PETS vs. 2.0): a patient-reported measure of treatment burden<br>Eton, D. T.,Lee, M. K.,St. Sauver, J. L.,Anderson, R. T.<br>Level: 2, State: Excluded	Level 2, Form level_2_full_text_screening, This study used the Burden of Treatment Theory: -> None of the above
89	RefID: 89, Deriving and validating a brief	Level 2, Form

	measure of treatment burden to assess person-centered healthcare quality in primary care: a multi-method study<br>Eton, D. T.,Linzer, M.,Boehm, D. H.,Vanderboom, C. E.,Rogers, E. A.,Frost, M. H.,Wambua, M.,Vang, M.,Poplau, S.,Lee, M. K.,Anderson, R. T.<br>Level: 2, State: Excluded	level_2_full_text_screening, This study used the Burden of Treatment Theory: -> None of the above
240	RefID: 240, Development and validation of the Patient Experience with Treatment and Self-management (PETS): a patient-reported measure of treatment burden<br>Eton, D. T.,Yost, K. J.,Lai, J. S.,Ridgeway, J. L.,Egginton, J. S.,Rosedahl, J. K.,Linzer, M.,Boehm, D. H.,Thakur, A.,Poplau, S.,Odell, L.,Montori, V. M.,May, C. R.,Anderson, R. T.<br>Level: 1, State: Excluded	Level 1, Form level_1__titleabstract_screeni
526	RefID: 526, Finalizing a measurement framework for the burden of treatment in complex patients with chronic conditions<br>Eton, David T.,Ridgeway, Jennifer L.,Egginton, Jason S.,Tiedje, Kristina,Linzer, Mark,Boehm, Deborah H.,Poplau, Sara,de Oliveira, Djenane Ramalho,Odell, Laura,Montori, Victor M.,May, Carl R.,Anderson, Roger T.<br>Level: 2, State: Excluded	Level 2, Form level_2_full_text_screening, This study used the Burden of Treatment Theory: -> None of the above
602	RefID: 602, Risk factors for hospital readmissions in pneumonia patients: A systematic review and meta-analysis<br>Fang, Y. Y.,Ni, J. C.,Wang, Y.,Yu, J. H.,Fu, L. L.<br>Level: 2, State: Excluded	Level 2, Form level_2_full_text_screening, This study used the Burden of Treatment Theory: -> None of the above
171	RefID: 171, Perspectives of Patients in Identifying Their Values-Based Health Priorities<br>Feder, S. L.,Kiwak, E.,Costello, D.,Dindo, L.,Hernandez-Bigos, K.,Vo, L.,Geda, M.,Blaum, C.,Tinetti, M. E.,Naik, A. D.<br>Level: 2, State: Excluded	Level 2, Form level_2_full_text_screening, This study used the Burden of Treatment Theory: -> None of the above
201	RefID: 201, Perspectives of Patients, Clinicians, and Health System Leaders on Changes Needed to Improve the Health Care and Outcomes of Older Adults With Multiple Chronic Conditions<br>Ferris, R.,Blaum, C.,Kiwak, E.,Austin, J.,Esterson, J.,Harkless, G.,Oftedahl, G.,Parchman, M.,Van Ness, P. H.,Tinetti, M.	Level 2, Form level_2_full_text_screening, This study used the Burden of Treatment Theory: -> None of the above

	E.<br>Level: 2, State: Excluded	
78	RefID: 78, ‘Just keep taking them, keep hoping they’ll work’: A qualitative study of adhering to medications for multimorbidity<br>Foley, L.,Hynes, L.,Murphy, A. W.,Molloy, G. J.<br>Level: 2, State: Excluded	Level 2, Form level_2_full_text_screening, This study used the Burden of Treatment Theory: -> None of the above
80	RefID: 80, Chronic condition self-management is a social practice<br>Franklin, M.,Willis, K.,Lewis, S.,Smith, L.<br>Level: 1, State: Excluded	Level 1, Form level_1__titleabstract_screeni
271	RefID: 271, The burden of Comorbidity in people with chronic kidney disease stage 3: A cohort study<br>Fraser, S. D. S.,Roderick, P. J.,May, C. R.,McIntyre, N.,McIntyre, C.,Fluck, R. J.,Shardlow, A.,Taal, M. W.<br>Level: 2, State: Excluded	Level 2, Form level_2_full_text_screening, This study used the Burden of Treatment Theory: -> None of the above
250	RefID: 250, Multimorbidity in people with chronic kidney disease: Implications for outcomes and treatment<br>Fraser, S. D. S.,Taal, M. W.<br>Level: 2, State: Excluded	Level 2, Form level_2_full_text_screening, This study used the Burden of Treatment Theory: -> None of the above
144	RefID: 144, Health literacy, multimorbidity, and patient-perceived treatment burden in individuals with cardiovascular disease. A Danish population-based study<br>Friis, K.,Lasgaard, M.,Pedersen, M. H.,Duncan, P.,Maindal, H. T.<br>Level: 2, State: Excluded	Level 2, Form level_2_full_text_screening, This study used the Burden of Treatment Theory: -> None of the above
238	RefID: 238, A qualitative investigation of lay perspectives of diagnosis and self-management strategies employed by people with progressive multiple sclerosis<br>Frost, J.,Grose, J.,Britten, N.<br>Level: 2, State: Excluded	Level 2, Form level_2_full_text_screening, This study used the Burden of Treatment Theory: -> None of the above
75	RefID: 75, Keeping in balance on the multimorbidity tightrope: A narrative analysis of older patients’ experiences of living with and managing multimorbidity<br>Fudge, N.,Swinglehurst, D.<br>Level: 2, State: Excluded	Level 2, Form level_2_full_text_screening, This study used the Burden of Treatment Theory: -> None of the above
617	RefID: 617, Keeping in balance on the multimorbidity tightrope: A narrative analysis of older patients’ experiences of living with and managing multimorbidity	Level 2, Form level_2_full_text_screening, This study used the Burden of Treatment Theory: -> None of the above

	Fudge, N.,Swinglehurst, D.<br>Level: 2, State: Excluded	
290	RefID: 290, Stroke, multimorbidity and polypharmacy in a nationally representative sample of 1,424,378 patients in Scotland: Implications for treatment burden<br>Gallacher, K. I.,Batty, G. D.,McLean, G.,Mercer, S. W.,Guthrie, B.,May, C. R.,Langhorne, P.,Mair, F. S.<br>Level: 2, State: Excluded	Level 2, Form level_2_full_text_screening, This study used the Burden of Treatment Theory: -> None of the above
214	RefID: 214, A conceptual model of treatment burden and patient capacity in stroke<br>Gallacher, K. I.,May, C. R.,Langhorne, P.,Mair, F. S.<br>Level: 2, State: Excluded	Level 2, Form level_2_full_text_screening, This study used the Burden of Treatment Theory: -> None of the above
145	RefID: 145, Systematic review of patient-reported measures of treatment burden in stroke<br>Gallacher, K. I.,Quinn, T.,Kidd, L.,Eton, D.,Dillon, M.,Elliot, J.,Johnston, N.,Erwin, P. J.,Mair, F.<br>Level: 2, State: Excluded	Level 2, Form level_2_full_text_screening, This study used the Burden of Treatment Theory: -> None of the above
130	RefID: 130, Value co-creation and its meaning for customers<br>Gallan, A. S.,Jefferies, J. G.<br>Level: 1, State: Excluded	Level 1, Form level_1__titleabstract_screeni
381	RefID: 381, Use of virtual consultations in an orthopaedic rehabilitation setting: how do changes in the work of being a patient influence patient preferences? A systematic review and qualitative synthesis<br>Gilbert, Anthony W.,Jones, Jeremy,Jaggi, Anju,May, Carl R.<br>Level: 2, State: Excluded	Level 2, Form level_2_full_text_screening, This study used the Burden of Treatment Theory: -> None of the above
259	RefID: 259, Do we have a clue: The treatment burden for the patient with cancer?<br>Given, B. A.,Given, C. W.,Vachon, E.,Hershey, D.<br>Level: 1, State: Excluded	Level 1, Form level_1__titleabstract_screeni
137	RefID: 137, Health-related preferences of older patients with multimorbidity: An evidence map<br>Gonzalez, A. I.,Schmucker, C.,Nothacker, J.,Motschall, E.,Nguyen, T. S.,Brueckle, M. S.,Blom, J.,Van Den Akker, M.,Röttger, K.,Wegwarth, O.,Hoffmann, T.,Straus, S. E.,Gerlach, F. M.,Meerpohl, J. J.,Muth, C.	Level 2, Form level_2_full_text_screening, This study used the Burden of Treatment Theory: -> None of the above

	Level: 2, State: Excluded	
234	RefID: 234, Burden of treatment in the light of the international classification of functioning, disability and health: a “best fit” framework synthesis<br>Gonçalves, A. C. V.,Jácome, C. I. O.,Demain, S. H.,Hunt, K. J.,Marques, A. S. P. D.<br>Level: 2, State: Excluded	Level 2, Form level_2_full_text_screening, This study used the Burden of Treatment Theory: -> None of the above
604	RefID: 604, Needs assessment for health service design for people with back pain in a hospital setting: A qualitative study<br>Gorgon, E.,Maka, K.,Kam, A.,Nisbet, G.,Sullivan, J.,Regan, G.,Pourkazemi, F.,Lin, J.,Mohamed, M.,Leaver, A.<br>Level: 2, State: Excluded	Level 2, Form level_2_full_text_screening, This study used the Burden of Treatment Theory: -> None of the above
177	RefID: 177, Uncovering the invisible patient work system through a case study of breast cancer self-management<br>Gorman, R. K.,Wellbeloved-Stone, C. A.,Valdez, R. S.<br>Level: 2, State: Excluded	Level 2, Form level_2_full_text_screening, This study used the Burden of Treatment Theory: -> None of the above
90	RefID: 90, Factors Associated With 30-Day Rehospitalization and Mortality in Older Patients After a Pneumonia Admission<br>Graversen, S. B.,Pedersen, H. S.,Sandbaek, A.,Foss, C. H.,Ribe, A. R.<br>Level: 2, State: Excluded	Level 2, Form level_2_full_text_screening, This study used the Burden of Treatment Theory: -> None of the above
169	RefID: 169, The NASSS Framework A Synthesis of Multiple Theories of Technology Implementation<br>Greenhalgh, T.,Abimbola, S.<br>Level: 1, State: Excluded	Level 1, Form level_1__titleabstract_screeni
277	RefID: 277, Six 'biases' against patients and carers in evidence-based medicine<br>Greenhalgh, T.,Snow, R.,Ryan, S.,Rees, S.,Salisbury, H.<br>Level: 1, State: Excluded	Level 1, Form level_1__titleabstract_screeni
222	RefID: 222, Beyond adoption: A new framework for theorizing and evaluating nonadoption, abandonment, and challenges to the scale-up, spread, and sustainability of health and care technologies<br>Greenhalgh, T.,Wherton, J.,Papoutsi, C.,Lynch, J.,Hughes, G.,A'Court, C.,Hinder, S.,Fahy, N.,Procter, R.,Shaw, S.<br>Level: 2, State: Excluded	Level 2, Form level_2_full_text_screening, This study used the Burden of Treatment Theory: -> None of the above
203	RefID: 203, Analysing the role of complexity	Level 2, Form

	in explaining the fortunes of technology programmes: Empirical application of the NASSS framework<br>Greenhalgh, T.,Wherton, J.,Papoutsi, C.,Lynch, J.,Hughes, G.,A'Court, C.,Hinder, S.,Procter, R.,Shaw, S.<br>Level: 2, State: Excluded	level_2_full_text_screening, This study used the Burden of Treatment Theory: -> None of the above
50	RefID: 50, Mixed methods process evaluation of my breathing matters, a digital intervention to support self-management of asthma<br>Greenwell, K.,Ainsworth, B.,Bruton, A.,Murray, E.,Russell, D.,Thomas, M.,Yardley, L.<br>Level: 2, State: Excluded	Level 2, Form level_2_full_text_screening, This study used the Burden of Treatment Theory: -> None of the above
73	RefID: 73, Taking charge of eczema self-management: A qualitative interview study with young people with eczema<br>Greenwell, K.,Ghio, D.,Muller, I.,Roberts, A.,McNiven, A.,Lawton, S.,Santer, M.<br>Level: 2, State: Excluded	Level 2, Form level_2_full_text_screening, This study used the Burden of Treatment Theory: -> None of the above
228	RefID: 228, A Systematic Review of Reviews Evaluating Technology-Enabled Diabetes Self-Management Education and Support<br>Greenwood, D. A.,Gee, P. M.,Fatkin, K. J.,Peeples, M.<br>Level: 1, State: Excluded	Level 1, Form level_1__titleabstract_screeni
103	RefID: 103, Iatrogenic dys-appearance: first-person accounts of chronic neuromuscular disease reveal unintended harms of treatment<br>Groven, K. S.,Braithwaite, J.,Dahl-Michelsen, T.<br>Level: 2, State: Excluded	Level 2, Form level_2_full_text_screening, This study used the Burden of Treatment Theory: -> None of the above
542	RefID: 542, Mediating role of psychological capital in the relationship between social support and treatment burden among older patients with chronic obstructive pulmonary disease<br>Gu, Jiaxin,Yang, Chaojuan,Zhang, Ke,Zhang, Qi<br>Level: 2, State: Excluded	Level 2, Form level_2_full_text_screening, This study used the Burden of Treatment Theory: -> None of the above
56	RefID: 56, Qualitative research methods and its application in nephrology<br>Guha, C.,Viecelli, A. K.,Wong, G.,Manera, K.,Tong, A.<br>Level: 2, State: Excluded	Level 2, Form level_2_full_text_screening, This study used the Burden of Treatment Theory: -> None of the above
215	RefID: 215, Challenges of Dealing with Financial Concerns during Life-Threatening	Level 2, Form level_2_full_text_screening, This

	Illness: Perspectives of Health Care Practitioners<br>Hageman, S. A.,Tarzian, A. J.,Cagle, J.<br>Level: 2, State: Excluded	study used the Burden of Treatment Theory: -> None of the above
119	RefID: 119, HIV positive and treated for cancer: The convergence of pressures “invisible” in HIV and “visible” in cancer<br>Hainsworth, E. G.,Shahmanesh, M.,Stevenson, F.<br>Level: 2, State: Excluded	Level 2, Form level_2_full_text_screening, This study used the Burden of Treatment Theory: -> None of the above
170	RefID: 170, Quality of Life and Value Assessment in Health Care<br>Hall, A.<br>Level: 2, State: Excluded	Level 2, Form level_2_full_text_screening, This study used the Burden of Treatment Theory: -> None of the above
373	RefID: 373, Quality of Life and Value Assessment in Health Care<br>Hall, Alicia<br>Level: 1, State: Excluded	Level 1, Form level_1__titleabstract_screeni
51	RefID: 51, Healthcare professionals’ perspective on treatment burden and patient capacity in low-income rural populations: challenges and opportunities<br>Hardman, R.,Begg, S.,Spelten, E.<br>Level: 2, State: Excluded	Level 2, Form level_2_full_text_screening, This study used the Burden of Treatment Theory: -> None of the above
61	RefID: 61, Multimorbidity and its effect on perceived burden, capacity and the ability to selfmanage in a low-income rural primary care population: A qualitative study<br>Hardman, R.,Begg, S.,Spelten, E.<br>Level: 2, State: Excluded	Level 2, Form level_2_full_text_screening, This study used the Burden of Treatment Theory: -> None of the above
195	RefID: 195, Care and capacities of human-centered design<br>Hargraves, I.<br>Level: 1, State: Excluded	Level 1, Form level_1__titleabstract_screeni
92	RefID: 92, Adherence and the Moral Construction of the Self: A Narrative Analysis of Anticoagulant Medication<br>Hawking, M. K. D.,Robson, J.,Taylor, S. J. C.,Swinglehurst, D.<br>Level: 2, State: Excluded	Level 2, Form level_2_full_text_screening, This study used the Burden of Treatment Theory: -> None of the above
244	RefID: 244, Person-centred medicines optimisation policy in England: An agenda for research on polypharmacy<br>Heaton, J.,Britten, N.,Krska, J.,Reeve, J.<br>Level: 1, State: Excluded	Level 1, Form level_1__titleabstract_screeni
270	RefID: 270, 'Rule your condition, don't let it	Level 2, Form

	rule you': Young adults' sense of mastery in their accounts of growing up with a chronic illness<br>Heaton, J.,Räisänen, U.,Salinas, M.<br>Level: 2, State: Excluded	level_2_full_text_screening, This study used the Burden of Treatment Theory: -> None of the above
151	RefID: 151, Patients’ Conceptualizations of Responsibility for Healthcare: A Typology for Understanding Differing Attributions in the Context of Patient Safety<br>Heavey, E.,Waring, J.,De Brún, A.,Dawson, P.,Scott, J.<br>Level: 2, State: Excluded	Level 2, Form level_2_full_text_screening, This study used the Burden of Treatment Theory: -> None of the above
86	RefID: 86, A framework for practical issues was developed to inform shared decision-making tools and clinical guidelines<br>Heen, A. F.,Vandvik, P. O.,Brandt, L.,Montori, V. M.,Lytvyn, L.,Guyatt, G.,Quinlan, C.,Agoritsas, T.<br>Level: 2, State: Excluded	Level 2, Form level_2_full_text_screening, This study used the Burden of Treatment Theory: -> None of the above
192	RefID: 192, Foot pain and foot health in an educated population of adults: Results from the Glasgow Caledonian University Alumni Foot Health Survey<br>Hendry, G. J.,Fenocchi, L.,Woodburn, J.,Steultjens, M.<br>Level: 1, State: Excluded	Level 1, Form level_1__titleabstract_screeni
96	RefID: 96, Ethical aspects of self-care: Comment on Riegel et al (2019) Self-care research: Where are we now? Where are we going?<br>Herber, O. R.,Krischel, M.,Whittal, A.<br>Level: 1, State: Excluded	Level 1, Form level_1__titleabstract_screeni
150	RefID: 150, Factors associated with patients' and GPs' assessment of the burden of treatment in multimorbid patients: A cross-sectional study in primary care<br>Herzig, L.,Zeller, A.,Pasquier, J.,Streit, S.,Neuner-Jehle, S.,Excoffier, S.,Haller, D. M.<br>Level: 2, State: Excluded	Level 2, Form level_2_full_text_screening, This study used the Burden of Treatment Theory: -> None of the above
221	RefID: 221, Aquatic therapy for boys with Duchenne muscular dystrophy (DMD): An external pilot randomised controlled trial<br>Hind, D.,Parkin, J.,Whitworth, V.,Rex, S.,Young, T.,Hampson, L.,Sheehan, J.,Maguire, C.,Cantrill, H.,Scott, E.,Epps, H.,Main, M.,Geary, M.,McMurchie, H.,Pallant, L.,Woods, D.,Freeman, J.,Lee, E.,Eagle, M.,Willis, T.,Muntoni, F.,Baxter, P.	Level 2, Form level_2_full_text_screening, This study used the Burden of Treatment Theory: -> None of the above

	Level: 2, State: Excluded	
242	RefID: 242, Aquatic therapy for children with duchenne muscular dystrophy: A pilot feasibility randomised controlled trial and mixed-methods process evaluation<br>Hind, D.,Parkin, J.,Whitworth, V.,Rex, S.,Young, T.,Hampson, L.,Sheehan, J.,Maguire, C.,Cantrill, H.,Scott, E.,Epps, H.,Main, M.,Geary, M.,McMurchie, H.,Pallant, L.,Woods, D.,Freeman, J.,Lee, E.,Eagle, M.,Willis, T.,Muntoni, F.,Baxter, P.<br>Level: 2, State: Excluded	Level 2, Form level_2_full_text_screening, This study used the Burden of Treatment Theory: -> None of the above
596	RefID: 596, Development of models of care coordination for rare conditions: A qualitative study<br>Holly Walton, Amy Simpson, Angus I.G. Ramsay, Amy Hunter, Jennifer Jones, Pei Li Ng, Kerry Leeson-Beevers, Lara Bloom, Joe Kai, Maria Kokocinska, Alastair G Sutcliffe, Stephen Morris, Naomi J. Fulop<br>Level: 2, State: Excluded	Level 2, Form level_2_full_text_screening, This study used the Burden of Treatment Theory: -> None of the above
597	RefID: 597, Developing a taxonomy of care coordination for people living with rare conditions: A qualitative study<br>Holly Walton, Amy Simpson, Angus I.G. Ramsay, Emma Hudson, Amy Hunter, Jennifer Jones, Pei Li Ng, Kerry Leeson-Beevers, Lara Bloom, Joe Kai, Larissa Kerecuk, Maria Kokocinska, Alastair G Sutcliffe, Stephen Morris, Naomi J Fulop<br>Level: 2, State: Excluded	Level 2, Form level_2_full_text_screening, This study used the Burden of Treatment Theory: -> None of the above
595	RefID: 595, Patients' and carers' experiences of, and engagement with remote home monitoring services for COVID-19 patients: a rapid mixed-methods study<br>Holly Walton, Cecilia Vindrola-Padros, Nadia Crellin, Manbinder S Sidhu, Lauren Herlitz, Ian Litchfield, Jo Ellins, Pei Li Ng, Efthalia Massou, Sonila M Tomini, Naomi J Fulop<br>Level: 2, State: Excluded	Level 2, Form level_2_full_text_screening, This study used the Burden of Treatment Theory: -> None of the above
431	RefID: 431, Structured lifestyle education to support weight loss for people with schizophrenia, schizoaffective disorder and first episode psychosis: the STEPWISE RCT<br>Holt, Richard Ig,Hind, Daniel,Gossage-Worrall, Rebecca,Bradburn, Michael J.,Saxon, David,McCrone, Paul,Morris, Tiyi A.,Etherington, Angela,Shiers, David,Barnard,	Level 2, Form level_2_full_text_screening, This study used the Burden of Treatment Theory: -> None of the above

	Katharine,Swaby, Lizzie,Edwardson, Charlotte,Carey, Marian E.,Davies, Melanie J.,Dickens, Christopher M.,Doherty, Yvonne,French, Paul,Greenwood, Kathryn E.,Kalidindi, Sridevi,Khunti, Kamlesh,Laugharne, Richard,Pendlebury, John,Rathod, Shanaya,Siddiqi, Najma,Wright, Stephen,Waller, Glenn,Gaughran, Fiona,Barnett, Janette,Northern, Alison<br>Level: 2, State: Excluded	
94	RefID: 94, Patient and health-care worker perspectives on the short-course regimen for treatment of drug-resistant tuberculosis in Karakalpakstan, Uzbekistan<br>Horter, S.,Achar, J.,Gray, N.,Parpieva, N.,Tigay, Z.,Singh, J.,Stringer, B.<br>Level: 2, State: Excluded	Level 2, Form level_2_full_text_screening, This study used the Burden of Treatment Theory: -> None of the above
53	RefID: 53, Change in treatment burden among people with multimorbidity: Protocol of a follow up survey and development of efficient measurement tools for primary care<br>Hounkpatin, H. O.,Roderick, P.,Morris, J. E.,Harris, S.,Watson, F.,Dambha-Miller, H.,Roberts, H.,Walsh, B.,Smith, D.,Fraser, S. D. S.,Crowe, S.,Compton, E.,Hughes, J.,Tan, Q.,Kelsey, M.,A. R. C. Wessex Treatment Burden Group<br>Level: 2, State: Excluded	Level 2, Form level_2_full_text_screening, This study used the Burden of Treatment Theory: -> None of the above
311	RefID: 311, Enabling patients in effective self-management of breathlessness in lung cancer: the neglected pillar of personalized medicine<br>Howell, Doris<br>Level: 1, State: Excluded	Level 1, Form level_1__titleabstract_screeni
63	RefID: 63, Feeling responsible: Family caregivers' attitudes and experiences of shared decision-making regarding people diagnosed with schizophrenia: A qualitative study<br>Huang, C.,Lam, L.,Plummer, V.,Cross, W. M.<br>Level: 2, State: Excluded	Level 2, Form level_2_full_text_screening, This study used the Burden of Treatment Theory: -> None of the above
123	RefID: 123, Application of normalisation process theory in understanding implementation processes in primary care settings in the UK: A systematic review<br>Huddlestone, L.,Turner, J.,Eborall, H.,Hudson, N.,Davies, M.,Martin, G.<br>Level: 1, State: Excluded	Level 1, Form level_1__titleabstract_screeni
106	RefID: 106, Men, chronic illness and healthwork: accounts from male partners of	Level 2, Form level_2_full_text_screening, This

	women with endometriosis<br>Hudson, N.,Law, C.,Culley, L.,Mitchell, H.,Denny, E.,Norton, W.,Raine-Fenning, N.<br>Level: 2, State: Excluded	study used the Burden of Treatment Theory: -> None of the above
199	RefID: 199, Symbolic, collective and intimate spaces: An ethnographic approach to the places of integrated care<br>Hughes, G.<br>Level: 1, State: Excluded	Level 1, Form level_1__titleabstract_screeni
620	RefID: 620, Dealing with being prescribed cardiovascular preventive medication: A narrative analysis of qualitative interviews with patients with recent acute coronary heart disease in Sweden<br>Hultberg, J.,Nilsson, S.,Rudebeck, C. E.,Köhler, A. K.<br>Level: 2, State: Excluded	Level 2, Form level_2_full_text_screening, This study used the Burden of Treatment Theory: -> None of the above
59	RefID: 59, Factors influencing treatment burden in colorectal cancer patients undergoing curative surgery: A cross-sectional study<br>Husebø, A. M. L.,Dalen, I.,Richardson, A.,Bru, E.,Søreide, J. A.<br>Level: 2, State: Excluded	Level 2, Form level_2_full_text_screening, This study used the Burden of Treatment Theory: -> None of the above
74	RefID: 74, Cancer-related fatigue and treatment burden in surgically treated colorectal cancer patients – A cross-sectional study<br>Husebø, A. M. L.,Dalen, I.,Søreide, J. A.,Bru, E.,Richardson, A.<br>Level: 2, State: Excluded	Level 2, Form level_2_full_text_screening, This study used the Burden of Treatment Theory: -> None of the above
112	RefID: 112, Health professionals' perceptions of colorectal cancer patients' treatment burden and their supportive work to ameliorate the burden - a qualitative study<br>Husebø, A. M. L.,Karlsen, B.,Husebø, S. E.<br>Level: 2, State: Excluded	Level 2, Form level_2_full_text_screening, This study used the Burden of Treatment Theory: -> None of the above
180	RefID: 180, The patient experience with treatment and self-management (PETS) questionnaire: Translation and cultural adaption of the Norwegian version<br>Husebø, A. M. L.,Morken, I. M.,Eriksen, K. S.,Nordfonn, O. K.<br>Level: 1, State: Excluded	Level 1, Form level_1__titleabstract_screeni
282	RefID: 282, Impact of periodontal status on oral health-related quality of life in patients with and without type 2 diabetes	Level 1, Form level_1__titleabstract_screeni

	Irani, F. C.,Wassall, R. R.,Preshaw, P. M.<br>Level: 1, State: Excluded	
69	RefID: 69, The self-management work of food hypersensitivity<br>Jakobsen, M. D.,Obstfelder, A.,Braaten, T.,Abelsen, B.<br>Level: 2, State: Excluded	Level 2, Form level_2_full_text_screening, This study used the Burden of Treatment Theory: -> None of the above
149	RefID: 149, What makes women with food hypersensitivity do self-management work?<br>Jakobsen, M. D.,Obstfelder, A.,Braaten, T.,Abelsen, B.<br>Level: 2, State: Excluded	Level 2, Form level_2_full_text_screening, This study used the Burden of Treatment Theory: -> None of the above
481	RefID: 481, Self-Reported Food Hypersensitivity: Prevalence, Characteristics, and Comorbidities in the Norwegian Women and Cancer Study<br>Jakobsen, Monika Dybdahl,Braaten, Tonje,Obstfelder, Aud,Abelsen, Birgit<br>Level: 1, State: Excluded	Level 1, Form level_1__titleabstract_screeni
82	RefID: 82, Unmet healthcare needs among midlife adults with mental distress and multiple chronic conditions<br>Johnson, P. J.,Mentzer, K. M.,Jou, J.,Upchurch, D. M.<br>Level: 1, State: Excluded	Level 1, Form level_1__titleabstract_screeni
618	RefID: 618, Unmet healthcare needs among midlife adults with mental distress and multiple chronic conditions<br>Johnson, P. J.,Mentzer, K. M.,Jou, J.,Upchurch, D. M.<br>Level: 2, State: Excluded	Level 2, Form level_2_full_text_screening, This study used the Burden of Treatment Theory: -> None of the above
133	RefID: 133, The development of a new questionnaire to measure the burden of immunoglobulin treatment in patients with primary immunodeficiencies: The IgBoT-35<br>Jones, G. L.,Williams, K.,Edmondson-Jones, M.,Prevot, J.,Drabwell, J.,Solis, L.,Shrimpton, A.,Mahlaoui, N.<br>Level: 2, State: Excluded	Level 2, Form level_2_full_text_screening, This study used the Burden of Treatment Theory: -> None of the above
486	RefID: 486, Safety of intravenous iron use in chronic kidney disease<br>Kalra, Philip A.,Bhandari, Sunil<br>Level: 1, State: Excluded	Level 1, Form level_1__titleabstract_screeni
181	RefID: 181, Theory and practice in digital behaviour change: A matrix framework for the co-production of digital services that engage, empower and emancipate marginalised people	Level 2, Form level_2_full_text_screening, This study used the Burden of Treatment Theory: -> None of the above

	living with complex and chronic conditions<br>Kayser, L.,Nøhr, C.,Bertelsen, P.,Botin, L.,Villumsen, S.,Showell, C.,Turner, P.<br>Level: 2, State: Excluded	
162	RefID: 162, Development of the multidimensional Readiness and Enablement Index for health Technology (READHY) tool to measure individuals’ health technology readiness: Initial testing in a cancer rehabilitation setting<br>Kayser, L.,Rossen, S.,Karnoe, A.,Elsworth, G.,Vibe-Petersen, J.,Christensen, J. F.,Ried-Larsen, M.,Osborne, R. H.<br>Level: 2, State: Excluded	Level 2, Form level_2_full_text_screening, This study used the Burden of Treatment Theory: -> None of the above
208	RefID: 208, Clinical encounter and the logic of relationality: Reconfiguring bodies and subjectivities in clinical relations<br>Kazimierczak, K. A.<br>Level: 2, State: Excluded	Level 2, Form level_2_full_text_screening, This study used the Burden of Treatment Theory: -> None of the above
105	RefID: 105, Exploring how virtual primary care visits affect patient burden of treatment<br>Kelley, L. T.,Phung, M.,Stamenova, V.,Fujioka, J.,Agarwal, P.,Onabajo, N.,Wong, I.,Nguyen, M.,Bhatia, R. S.,Bhattacharyya, O.<br>Level: 2, State: Excluded	Level 2, Form level_2_full_text_screening, This study used the Burden of Treatment Theory: -> None of the above
129	RefID: 129, Opportunity or Burden? A Behavioral Framework for Patient Engagement<br>Kimerling, R.,Lewis, E. T.,Javier, S. J.,Zulman, D. M.<br>Level: 2, State: Excluded	Level 2, Form level_2_full_text_screening, This study used the Burden of Treatment Theory: -> None of the above
194	RefID: 194, 'More constricting than inspiring' - GPs find chronic care programmes of limited clinical utility. A qualitative study<br>Kristensen, M. A. T.,Due, T. D.,Hølge-Hazelton, B.,Guassora, A. D.,Waldorff, F. B.<br>Level: 2, State: Excluded	Level 2, Form level_2_full_text_screening, This study used the Burden of Treatment Theory: -> None of the above
220	RefID: 220, How general practitioners perceive and assess self-care in patients with multiple chronic conditions: A qualitative study<br>Kristensen, M. A. T.,Hølge-Hazelton, B.,Waldorff, F. B.,Guassora, A. D.<br>Level: 2, State: Excluded	Level 2, Form level_2_full_text_screening, This study used the Burden of Treatment Theory: -> None of the above
54	RefID: 54, Patient administrative burden in the US health care system<br>Kyle, M. A.,Frakt, A. B.	Level 2, Form level_2_full_text_screening, This study used the Burden of Treatment

	Level: 2, State: Excluded	Theory: -> None of the above
444	RefID: 444, Towards a More Patient-Centered Approach to Medication Safety<br>Lee, Joy L.,Dy, Sydney M.,Gurses, Ayse P.,Kim, Julia M.,Suarez-Cuervo, Catalina,Berger, Zackary D.,Brown, Rachel,Xiao, Yan<br>Level: 1, State: Excluded	Level 1, Form level_1__titleabstract_screeni
285	RefID: 285, Minimally disruptive medicine: A pragmatically comprehensive model for delivering care to patients with multiple chronic conditions<br>Leppin, A. L.,Montori, V. M.,Gionfriddo, M. R.<br>Level: 1, State: Excluded	Level 1, Form level_1__titleabstract_screeni
85	RefID: 85, Decreasing patient-reported burden of treatment: A systematic review of quantitative interventional studies<br>Lesage, A.,Leclère, B.,Moret, L.,Le Glatin, C.<br>Level: 2, State: Excluded	Level 2, Form level_2_full_text_screening, This study used the Burden of Treatment Theory: -> None of the above
141	RefID: 141, Patient experience after kidney transplant: a conceptual framework of treatment burden<br>Lorenz, E. C.,Egginton, J. S.,Stegall, M. D.,Cheville, A. L.,Heilman, R. L.,Nair, S. S.,Mai, M. L.,Eton, D. T.<br>Level: 2, State: Excluded	Level 2, Form level_2_full_text_screening, This study used the Burden of Treatment Theory: -> None of the above
60	RefID: 60, What is the impact of day care on older people with long-term conditions: A systematic review<br>Lunt, C.,Dowrick, C.,Lloyd-Williams, M.<br>Level: 2, State: Excluded	Level 2, Form level_2_full_text_screening, This study used the Burden of Treatment Theory: -> None of the above
173	RefID: 173, Usefulness of wearable cameras as a tool to enhance chronic disease self-management: Scoping review<br>Maddison, R.,Cartledge, S.,Rogerson, M.,Goedhart, N. S.,Ragbir Singh, T.,Neil, C.,Phung, D.,Ball, K.<br>Level: 2, State: Excluded	Level 2, Form level_2_full_text_screening, This study used the Burden of Treatment Theory: -> None of the above
237	RefID: 237, Multimorbidity: What next?<br>Mair, F. S.,Gallacher, K. I.<br>Level: 1, State: Excluded	Level 1, Form level_1__titleabstract_screeni
527	RefID: 527, Thinking about the burden of treatment<br>Mair, Frances S.,May, Carl R.<br>Level: 1, State: Excluded	Level 1, Form level_1__titleabstract_screeni

576	RefID: 576, Dose Orchestration and System Enhancement (DOSE): A practice model based on the Habituation-Intention Framework<br>Mansukhani, Sonal,Kieser, Mara,Ricci, Daniel,Chewning, Betty<br>Level: 2, State: Excluded	Level 2, Form level_2_full_text_screening, This study used the Burden of Treatment Theory: -> None of the above
268	RefID: 268, Chronic obstructive pulmonary disease: Outcome measures of respiratory physiotherapy in stable and acute periods<br>Marques, A.,Oliveira, A.,Cruz, J.,Jácome, C.<br>Level: 1, State: Excluded	Level 1, Form level_1__titleabstract_screeni
196	RefID: 196, An assessment of patient burdens from head and neck cancer survivorship care<br>Massa, S. T.,Rohde, R. L.,McKinstry, C.,Gresham, M.,Osazuwa-Peters, N.,Ward, G. M.,Walker, R. J.<br>Level: 2, State: Excluded	Level 2, Form level_2_full_text_screening, This study used the Burden of Treatment Theory: -> None of the above
95	RefID: 95, Narrative inquiries from kidney transplant patients: From the onset of the disease to the transplant<br>Mathilde, N.,Catherine, P.,Pascale, V.,Xavier, G.<br>Level: 2, State: Excluded	Level 2, Form level_2_full_text_screening, This study used the Burden of Treatment Theory: -> None of the above
209	RefID: 209, A qualitative study on the experiences and perspectives of public sector patients in Cape Town in managing the workload of demands of HIV and type 2 diabetes multimorbidity<br>Matima, R.,Murphy, K.,Levitt, N. S.,BeLue, R.,Oni, T.<br>Level: 2, State: Excluded	Level 2, Form level_2_full_text_screening, This study used the Burden of Treatment Theory: -> None of the above
284	RefID: 284, Making sense of technology adoption in healthcare: Meso-level considerations<br>May, C. R.<br>Level: 1, State: Excluded	Level 1, Form level_1__titleabstract_screeni
200	RefID: 200, Using Normalization Process Theory in feasibility studies and process evaluations of complex healthcare interventions: A systematic review<br>May, C. R.,Cummings, A.,Girling, M.,Bracher, M.,Mair, F. S.,May, C. M.,Murray, E.,Myall, M.,Rapley, T.,Finch, T.<br>Level: 1, State: Excluded	Level 1, Form level_1__titleabstract_screeni
503	RefID: 503, Experiences of long-term life-limiting conditions among patients and carers: what can we learn from a meta-review of	Level 2, Form level_2_full_text_screening, This study used the Burden of Treatment

	systematic reviews of qualitative studies of chronic heart failure, chronic obstructive pulmonary disease and chronic kidney disease?<br>May, Carl R.,Cummings, Amanda,Myall, Michelle,Harvey, Jonathan,Pope, Catherine,Griffiths, Peter,Roderick, Paul,Arber, Mick,Boehmer, Kasey,Mair, Frances S.,Richardson, Alison<br>Level: 2, State: Excluded	Theory: -> None of the above
532	RefID: 532, Rethinking the patient: using Burden of Treatment Theory to understand the changing dynamics of illness<br>May, Carl R.,Eton, David T.,Boehmer, Kasey,Gallacher, Katie,Hunt, Katherine,MacDonald, Sara,Mair, Frances S.,May, Christine M.,Montori, Victor M.,Richardson, Alison,Rogers, Anne E.,Shippee, Nathan<br>Level: 1, State: Excluded	Level 1, Form level_1__titleabstract_screeni
489	RefID: 489, Implementation, context and complexity<br>May, Carl R.,Johnson, Mark,Finch, Tracy<br>Level: 1, State: Excluded	Level 1, Form level_1__titleabstract_screeni
100	RefID: 100, Patients' experiences of using smartphone apps to support self-management and improve medication adherence in hypertension: Qualitative study<br>McBride, C. M.,Morrissey, E. C.,Molloy, G. J.<br>Level: 2, State: Excluded	Level 2, Form level_2_full_text_screening, This study used the Burden of Treatment Theory: -> None of the above
488	RefID: 488, Self-monitoring and self-management: new interventions to improve blood pressure control<br>McCartney, David E.,McManus, Richard J.<br>Level: 1, State: Excluded	Level 1, Form level_1__titleabstract_screeni
124	RefID: 124, Informal care management after traumatic brain injury: perspectives on informal carer workload and capacity<br>McIntyre, M.,Ehrlich, C.,Kendall, E.<br>Level: 2, State: Excluded	Level 2, Form level_2_full_text_screening, This study used the Burden of Treatment Theory: -> None of the above
160	RefID: 160, The social networks of New Zealand patients with multimorbidity and the work of those nominated as their 'significant supporters': An exploratory study<br>McKinlay, E.,McDonald, J.,Darlow, B.,Perry, M.<br>Level: 2, State: Excluded	Level 2, Form level_2_full_text_screening, This study used the Burden of Treatment Theory: -> None of the above

262	RefID: 262, Multimorbidity in chronic disease: Impact on health care resources and costs<br>McPhail, S. M.<br>Level: 1, State: Excluded	Level 1, Form level_1__titleabstract_screeni
104	RefID: 104, Experiences with and lessons learned from developing, implementing, and evaluating a support program for older hearing aid users and their communication partners in the hearing aid dispensing setting<br>Meijerink, J. F. J.,Pronk, M.,Kramer, S. E.<br>Level: 1, State: Excluded	Level 1, Form level_1__titleabstract_screeni
111	RefID: 111, A cross-sectional study of Swiss ambulatory care services use by multimorbid patients in primary care in the light of the Andersen model<br>Messi, M.,Mueller, Y.,Haller, D. M.,Zeller, A.,Neuner-Jehle, S.,Streit, S.,Burnand, B.,Herzig, L.<br>Level: 2, State: Excluded	Level 2, Form level_2_full_text_screening, This study used the Burden of Treatment Theory: -> None of the above
72	RefID: 72, Burden of Treatment Among Older Adults With Newly Diagnosed Multiple Myeloma<br>Mian, H. S.,Fiala, M. A.,Wildes, T. M.<br>Level: 2, State: Excluded	Level 2, Form level_2_full_text_screening, This study used the Burden of Treatment Theory: -> None of the above
107	RefID: 107, A qualitative multicase study of the trajectories of prolonged critical illness: Patient, family, and healthcare professionals' experiences<br>Minton, C.,Batten, L.,Huntington, A.<br>Level: 2, State: Excluded	Level 2, Form level_2_full_text_screening, This study used the Burden of Treatment Theory: -> None of the above
216	RefID: 216, The effect of individual and mixed rewards on diabetes management: A feasibility randomized controlled trial [version 2; referees: 2 approved]<br>Miranda, J. J.,Lazo-Porras, M.,Bernabe-Ortiz, A.,Pesantes, M. A.,Diez-Canseco, F.,Cornejo, S. P.,Trujillo, A. J.<br>Level: 1, State: Excluded	Level 1, Form level_1__titleabstract_screeni
172	RefID: 172, The effect of individual and mixed rewards on diabetes management: A feasibility randomized controlled trial [version 3; referees: 2 approved]<br>Miranda, J. J.,Lazo-Porras, M.,Bernabe-Ortiz, A.,Pesantes, M. A.,Diez-Canseco, F.,Cornejo, S. P.,Trujillo, A. J.<br>Level: 2, State: Excluded	Level 2, Form level_2_full_text_screening, This study used the Burden of Treatment Theory: -> None of the above

283	RefID: 283, Waving not drowning: Virtue ethics in general practice<br>Misselbrook, D.<br>Level: 1, State: Excluded	Level 1, Form level_1__titleabstract_screeni
279	RefID: 279, Editorials: Clinical decision making in dementia: Mapping the minefields<br>Misselbrook, D.<br>Level: 1, State: Excluded	Level 1, Form level_1__titleabstract_screeni
515	RefID: 515, Clinical decision making in dementia: mapping the minefields<br>Misselbrook, David<br>Level: 1, State: Excluded	Level 1, Form level_1__titleabstract_screeni
584	RefID: 584, Aristotle, Hume and the goals of medicine<br>Misselbrook, David<br>Level: 1, State: Excluded	Level 1, Form level_1__titleabstract_screeni
235	RefID: 235, Current and future perspectives on the management of polypharmacy<br>Molokhia, M.,Majeed, A.<br>Level: 2, State: Excluded	Level 2, Form level_2_full_text_screening, This study used the Burden of Treatment Theory: -> None of the above
249	RefID: 249, Big Science: Research Collaboration for Evidence-Based Care<br>Montori, V. M.<br>Level: 1, State: Excluded	Level 1, Form level_1__titleabstract_screeni
305	RefID: 305, Removing the blindfold: The centrality of care in caring for patients with multiple chronic conditions<br>Montori, Victor M.<br>Level: 1, State: Excluded	Level 1, Form level_1__titleabstract_screeni
622	RefID: 622, Experiences of recovery and a new care pathway for people with pain after total knee replacement: qualitative research embedded in the STAR trial<br>Moore, A.,Wylde, V.,Bruce, J.,Howells, N.,Bertram, W.,Eccleston, C.,Gooberman-Hill, R.<br>Level: 2, State: Excluded	Level 2, Form level_2_full_text_screening, This study used the Burden of Treatment Theory: -> None of the above
589	RefID: 589, Context and complexity: the meaning of self-management for older adults with heart disease<br>Moore, Lucy,Frost, Julia,Britten, Nicky<br>Level: 2, State: Excluded	Level 2, Form level_2_full_text_screening, This study used the Burden of Treatment Theory: -> None of the above
135	RefID: 135, Resistance or appropriation?: Uptake of exercise after a nurse-led intervention to promote self-management for osteoarthritis	Level 2, Form level_2_full_text_screening, This study used the Burden of Treatment Theory: -> None of the above

	Morden, A.,Ong, B. N.,Jinks, C.,Healey, E.,Finney, A.,Dziedzic, K. S.<br>Level: 2, State: Excluded	
606	RefID: 606, Resistance or appropriation?: Uptake of exercise after a nurse-led intervention to promote self-management for osteoarthritis<br>Morden, A.,Ong, B. N.,Jinks, C.,Healey, E.,Finney, A.,Dziedzic, K. S.<br>Level: 2, State: Excluded	Level 2, Form level_2_full_text_screening, This study used the Burden of Treatment Theory: -> None of the above
590	RefID: 590, Setting a research agenda for medical overuse<br>Morgan, Daniel,Brownlee, Shannon,Leppin, Aaron,Kressin, Nancy,Dhruva, Sanket,Levin, Les,Landon, Bruce,Zezza, Mark,Schmidt, Harald,Saini, Vikas,Elshaug, Adam<br>Level: 1, State: Excluded	Level 1, Form level_1__titleabstract_screeni
491	RefID: 491, Evolving 'self'-management: exploring the role of social network typologies on individual long-term condition management<br>Morris, Rebecca L.,Kennedy, Anne,Sanders, Caroline<br>Level: 2, State: Excluded	Level 2, Form level_2_full_text_screening, This study used the Burden of Treatment Theory: -> None of the above
202	RefID: 202, Smartphone apps for improving medication adherence in hypertension: Patients' perspectives<br>Morrissey, E. C.,Casey, M.,Glynn, L. G.,Walsh, J. C.,Molloy, G. J.<br>Level: 2, State: Excluded	Level 2, Form level_2_full_text_screening, This study used the Burden of Treatment Theory: -> None of the above
204	RefID: 204, Qualitative process study to explore the perceived burdens and benefits of a digital intervention for self-managing high blood pressure in Primary Care in the UK<br>Morton, K.,Dennison, L.,Bradbury, K.,Band, R. J.,May, C.,Raftery, J.,Little, P.,McManus, R. J.,Yardley, L.<br>Level: 2, State: Excluded	Level 2, Form level_2_full_text_screening, This study used the Burden of Treatment Theory: -> None of the above
607	RefID: 607, Development, deployment and evaluation of digitally enabled, remote, supported rehabilitation for people with long COVID-19 (Living With COVID-19 Recovery): protocol for a mixed-methods study<br>Murray, E.,Goodfellow, H.,Bindman, J.,Blandford, A.,Bradbury, K.,Chaudhry, T.,Fernandez-Reyes, D.,Gomes, M.,Hamilton, F. L.,Heightman, M.,Henley, W.,Hurst, J.	Level 2, Form level_2_full_text_screening, This study used the Burden of Treatment Theory: -> None of the above

	R.,Hylton, H.,Linke, S.,Pfeffer, P.,Ricketts, W.,Robson, C.,Singh, R.,Stevenson, F. A.,Walker, S.,Waywell, J.<br>Level: 2, State: Excluded	
272	RefID: 272, RESTORE: An exploratory trial of a web-based intervention to enhance self-management of cancer-related fatigue: Findings from a qualitative process evaluation<br>eHealth/ telehealth/ mobile health systems<br>Myall, M.,May, C. R.,Grimmett, C.,May, C. M.,Calman, L.,Richardson, A.,Foster, C. L.<br>Level: 2, State: Excluded	Level 2, Form level_2_full_text_screening, This study used the Burden of Treatment Theory: -> None of the above
422	RefID: 422, Estrategias para promover la alfabetización en salud desde la atención primaria: una perspectiva que considera las realidades de los países de ingresos medios y bajos<br>Málaga, German,Cuba-Fuentes, María Sofía,Rojas-Mezarina, Leonardo,Romero-Albino, Zoila,Hamb, Alexandra,Paz-Soldán, Valerie A.<br>Level: 1, State: Excluded	Level 1, Form level_1__titleabstract_screeni
152	RefID: 152, Role of age and health in perceptions of returning to work: A qualitative study<br>Neary, J.,Katikireddi, S. V.,Brown, J.,Macdonald, E. B.,Thomson, H.<br>Level: 2, State: Excluded	Level 2, Form level_2_full_text_screening, This study used the Burden of Treatment Theory: -> None of the above
229	RefID: 229, Patient–provider concordance in the perception of illness and disease: A cross-sectional study among multimorbid patients and their general practitioners in Switzerland<br>Neuner-Jehle, S.,Zechmann, S.,Maissen, D. G.,Rosemann, T.,Senn, O.<br>Level: 2, State: Excluded	Level 2, Form level_2_full_text_screening, This study used the Burden of Treatment Theory: -> None of the above
167	RefID: 167, Closer is not better. Distance and proximity in the use of health care by women living with HIV and AIDS in Ouagadougou (Burkina Faso)<br>Nikiema, A.,Bonnet, E.,Tougma, A.,Marcis, F. L.<br>Level: 1, State: Excluded	Level 1, Form level_1__titleabstract_screeni
377	RefID: 377, Achieving person-centred health systems: levers and strategies<br>Nolte, Ellen,Anell, Anders<br>Level: 1, State: Excluded	Level 1, Form level_1__titleabstract_screeni
378	RefID: 378, The person at the centre? The role	Level 1, Form

	of self-management and self-management support<br>Nolte, Ellen,Anell, Anders<br>Level: 1, State: Excluded	level_1__titleabstract_screeni
67	RefID: 67, Burden of treatment in patients with chronic heart failure – A cross-sectional study<br>Nordfonn, O. K.,Morken, I. M.,Bru, L. E.,Larsen, A. I.,Husebø, A. M. L.<br>Level: 2, State: Excluded	Level 2, Form level_2_full_text_screening, This study used the Burden of Treatment Theory: -> None of the above
264	RefID: 264, Realizing the potential of patient engagement: Designing IT to support health in everyday life<br>Novak, L. L.,Unertl, K. M.,Holden, R. J.<br>Level: 2, State: Excluded	Level 2, Form level_2_full_text_screening, This study used the Burden of Treatment Theory: -> None of the above
76	RefID: 76, Burden of cancer trial participation: A qualitative sub-study of the INTERIM feasibility RCT<br>Nwolise, C.,Corrie, P.,Fitzpatrick, R.,Gupta, A.,Jenkinson, C.,Middleton, M.,Matin, R.<br>Level: 2, State: Excluded	Level 2, Form level_2_full_text_screening, This study used the Burden of Treatment Theory: -> None of the above
188	RefID: 188, Design hotspots for care of discordant chronic comorbidities: Patients' perspectives<br>Ongwere, T.,Cantor, G.,Martin, S. R.,Shih, P. C.,Clawson, J.,Connelly, K.<br>Level: 2, State: Excluded	Level 2, Form level_2_full_text_screening, This study used the Burden of Treatment Theory: -> None of the above
186	RefID: 186, Principle of rational prescribing and deprescribing in older adults with multiple chronic conditions<br>Ouellet, G. M.,Ouellet, J. A.,Tinetti, M. E.<br>Level: 2, State: Excluded	Level 2, Form level_2_full_text_screening, This study used the Burden of Treatment Theory: -> None of the above
108	RefID: 108, Adverse Childhood Experiences and Decreased Renal Function: Impact on All-Cause Mortality in U.S. Adults<br>Ozieh, M. N.,Garacci, E.,Campbell, J. A.,Walker, R. J.,Egede, L. E.<br>Level: 2, State: Excluded	Level 2, Form level_2_full_text_screening, This study used the Burden of Treatment Theory: -> None of the above
98	RefID: 98, The challenges of caring for children who require complex medical care at home: 'The go between for everyone is the parent and as the parent that's an awful lot of responsibility'<br>Page, B. F.,Hinton, L.,Harrop, E.,Vincent, C.<br>Level: 2, State: Excluded	Level 2, Form level_2_full_text_screening, This study used the Burden of Treatment Theory: -> None of the above
193	RefID: 193, A room with a view: a metaphor analysis of Vietnamese women's	Level 1, Form level_1__titleabstract_screeni

	representations of living with depression using photo elicitation<br>Palmer, V. J.,Furler, J.<br>Level: 1, State: Excluded	
66	RefID: 66, Interrogating the promise of technology in epilepsy care: systematic, hermeneutic review<br>Papoutsi, C.,Collins, C. D. E.,Christopher, A.,Shaw, S. E.,Greenhalgh, T.<br>Level: 2, State: Excluded	Level 2, Form level_2_full_text_screening, This study used the Burden of Treatment Theory: -> None of the above
612	RefID: 612, Danish validation of the Multimorbidity Treatment Burden Questionnaire (MTBQ) and findings from a population health survey: a mixed-methods study<br>Pedersen, M. H.,Duncan, P.,Lasgaard, M.,Friis, K.,Salisbury, C.,Breinholt Larsen, F.<br>Level: 1, State: Excluded	Level 1, Form level_1__titleabstract_screeni
217	RefID: 217, Chronicity and aging in cardiology: Geriatric cardiology, cardiac rehabilitation or care-related cardiogeriatric rehabilitation?<br>Pedretti, R. F. E.<br>Level: 1, State: Excluded	Level 1, Form level_1__titleabstract_screeni
127	RefID: 127, From geriatric cardiology to ‘cardio-geriatric’ prevention and rehabilitation: Need for a new core curriculum?<br>Pedretti, R. F. E.,Ambrosetti, M.,Sarzi Braga, S.<br>Level: 1, State: Excluded	Level 1, Form level_1__titleabstract_screeni
166	RefID: 166, Blood pressure control and burden of treatment in South African primary healthcare: A cross-sectional study<br>Pender, K.,Omole, O.<br>Level: 2, State: Excluded	Level 2, Form level_2_full_text_screening, This study used the Burden of Treatment Theory: -> None of the above
131	RefID: 131, Health and Wellness Coaching in Serving the Needs of Today’s Patients: A Primer for Healthcare Professionals<br>Perlman, A. I.,Abu Dabrh, A. M.<br>Level: 1, State: Excluded	Level 1, Form level_1__titleabstract_screeni
275	RefID: 275, Resilience in Vulnerable Populations With Type 2 Diabetes Mellitus and Hypertension: A Systematic Review and Meta-analysis<br>Pesantes, M. A.,Lazo-Porras, M.,Abu Dabrh, A. M.,Ávila-Ramírez, J. R.,Caycho,	Level 1, Form level_1__titleabstract_screeni

	M.,Villamonte, G. Y.,Sánchez-Pérez, G. P.,Málaga, G.,Bernabé-Ortiz, A.,Miranda, J. J.<br>Level: 1, State: Excluded	
159	RefID: 159, “It is not easy living with this illness”: A syndemic approach to medication adherence and lifestyle change among low-income diabetes patients in Lima, Peru<br>Pesantes, M. A.,Tetens, A.,Del Valle, A.,Miranda, J. J.<br>Level: 2, State: Excluded	Level 2, Form level_2_full_text_screening, This study used the Burden of Treatment Theory: -> None of the above
179	RefID: 179, “Experiences of the burden of treatment”—Patient reports of facilitated subcutaneous immunoglobulin treatment in adults with immunodeficiency<br>Petersson, C.,Fust, R.,Hagstedt, C.,Wågström, P.,Nilsdotter-Augustinsson,<br>Level: 2, State: Excluded	Level 2, Form level_2_full_text_screening, This study used the Burden of Treatment Theory: -> None of the above
613	RefID: 613, Experiences of psychological interventions in neurodegenerative diseases: a systematic review and thematic synthesis<br>Pinto, C.,Geraghty, A. W. A.,McLoughlin, C.,Pagnini, F.,Yardley, L.,Dennison, L.<br>Level: 2, State: Excluded	Level 2, Form level_2_full_text_screening, This study used the Burden of Treatment Theory: -> None of the above
579	RefID: 579, Treatment Burden of Medicare Beneficiaries With Stage I Non-Small-Cell Lung Cancer<br>Presley, Carolyn,Soulos, Pamela,Tinetti, Mary,Montori, Victor,Yu, James,Gross, Cary<br>Level: 2, State: Excluded	Level 2, Form level_2_full_text_screening, This study used the Burden of Treatment Theory: -> None of the above
48	RefID: 48, The work of managing multiple myeloma and its implications for treatment-related decision making: a qualitative study of patient and caregiver experiences<br>Pritlove, C.,Jassi, M.,Burns, B.,McCurdy, A.<br>Level: 2, State: Excluded	Level 2, Form level_2_full_text_screening, This study used the Burden of Treatment Theory: -> None of the above
158	RefID: 158, “It’s Hard Work”: A Feminist Political Economy Approach to Reconceptualizing “Work” in the Cancer Context<br>Pritlove, C.,Safai, P.,Angus, J. E.,Armstrong, P.,Jones, J. M.,Parsons, J.<br>Level: 2, State: Excluded	Level 2, Form level_2_full_text_screening, This study used the Burden of Treatment Theory: -> None of the above
64	RefID: 64, OPEN ARCH integrated care model: Experiences of older Australians and their carers<br>Quigley, R.,Russell, S.,Harvey, D.,Mann, J.<br>Level: 2, State: Excluded	Level 2, Form level_2_full_text_screening, This study used the Burden of Treatment Theory: -> None of the above

136	RefID: 136, Chronic conditions and behavioural change approaches to medication adherence: rethinking clinical guidance and recommendations<br>Read, S.,Morgan, J.,Gillespie, D.,Nollett, C.,Weiss, M.,Allen, D.,Anderson, P.,Waterman, H.<br>Level: 2, State: Excluded	Level 2, Form level_2_full_text_screening, This study used the Burden of Treatment Theory: -> None of the above
580	RefID: 580, Practical multidisciplinary approaches to heart failure management for improved patient outcome<br>Riley, Jillian,Masters, Jayne<br>Level: 2, State: Excluded	Level 2, Form level_2_full_text_screening, This study used the Burden of Treatment Theory: -> None of the above
459	RefID: 459, Chronicity and aging in Cardiology: Geriatric Cardiology, Cardiac Rehabilitation or Cardiogeriatric rehabilitation of related care?<br>Roberto, F. E.<br>Level: 1, State: Excluded	Level 1, Form level_1__titleabstract_screeni
236	RefID: 236, Patient-experienced burden of treatment in patients with multimorbidity – A systematic review of qualitative data<br>Rosbach, M.,Andersen, J. S.<br>Level: 2, State: Excluded	Level 2, Form level_2_full_text_screening, This study used the Burden of Treatment Theory: -> None of the above
183	RefID: 183, Burden of treatment in the face of childhood cancer: A quantitative study using medical records of deceased children<br>Rost, M.,Wangmo, T.,Rakic, M.,Acheson, E.,Rischewski, J.,Hengartner, H.,Kühne, T.,Elger, B. S.<br>Level: 2, State: Excluded	Level 2, Form level_2_full_text_screening, This study used the Burden of Treatment Theory: -> None of the above
114	RefID: 114, Why managing sciatica is difficult: Patients' experiences of an NHS sciatica pathway. A qualitative, interpretative study<br>Ryan, C.,Pope, C. J.,Roberts, L.<br>Level: 2, State: Excluded	Level 2, Form level_2_full_text_screening, This study used the Burden of Treatment Theory: -> None of the above
77	RefID: 77, Sharing the burden of treatment navigation: social work and the experiences of unhoused women in accessing health services in Santa Cruz<br>Saharan, A.,Balachander, M.,Sparke, M.<br>Level: 2, State: Excluded	Level 2, Form level_2_full_text_screening, This study used the Burden of Treatment Theory: -> None of the above
65	RefID: 65, Burden of treatment among elderly patients with cancer: A scoping review<br>Sav, A.,McMillan, S. S.,Akosile, A.<br>Level: 2, State: Excluded	Level 2, Form level_2_full_text_screening, This study used the Burden of Treatment Theory: -> None of the above

125	RefID: 125, The assessment and management of quality of life of older adults with diabetes mellitus<br>Sayyed Kassem, L.,Aron, D. C.<br>Level: 2, State: Excluded	Level 2, Form level_2_full_text_screening, This study used the Burden of Treatment Theory: -> None of the above
44	RefID: 44, Burden of mental health symptoms and perceptions of their management in in-centre hemodialysis care: a mixed methods study<br>Schick-Makaroff, K.,Wozniak, L. A.,Short, H.,Davison, S. N.,Klarenbach, S.,Buzinski, R.,Walsh, M.,Johnson, J. A.<br>Level: 2, State: Excluded	Level 2, Form level_2_full_text_screening, This study used the Burden of Treatment Theory: -> None of the above
223	RefID: 223, Minimally Disruptive Medicine for Patients with Diabetes<br>Serrano, V.,Spencer-Bonilla, G.,Boehmer, K. R.,Montori, V. M.<br>Level: 2, State: Excluded	Level 2, Form level_2_full_text_screening, This study used the Burden of Treatment Theory: -> None of the above
288	RefID: 288, Attaining minimally disruptive medicine: Context, challenges and a roadmap for implementation<br>Shippee, N. D.,Allen, S. V.,Leppin, A. L.,May, C. R.,Montori, V. M.<br>Level: 1, State: Excluded	Level 1, Form level_1__titleabstract_screeni
116	RefID: 116, The value of the Versus Arthritis, Nurse and Allied Health Professional Internship Scheme on early musculoskeletal career researchers: a short report from an intern's perspective<br>Simkins, J. M.,Healey, E. L.,Dziedzic, K. S.,Finney, A. G.<br>Level: 1, State: Excluded	Level 1, Form level_1__titleabstract_screeni
156	RefID: 156, Caregivers' experiences of a home support program after the hospital discharge of an older family member: A qualitative analysis<br>Slatyer, S.,Aoun, S. M.,Hill, K. D.,Walsh, D.,Whitty, D.,Toye, C.<br>Level: 2, State: Excluded	Level 2, Form level_2_full_text_screening, This study used the Burden of Treatment Theory: -> None of the above
45	RefID: 45, Interventions for improving outcomes in patients with multimorbidity in primary care and community setting: a systematic review<br>Smith, S. M.,Wallace, E.,Clyne, B.,Boland, F.,Fortin, M.<br>Level: 2, State: Excluded	Level 2, Form level_2_full_text_screening, This study used the Burden of Treatment Theory: -> None of the above
140	RefID: 140, A review of interventions to	Level 2, Form

	reduce pain in chronic wounds<br>Snelgrove, H.,Baileff, A.<br>Level: 2, State: Excluded	level_2_full_text_screening, This study used the Burden of Treatment Theory: -> None of the above
153	RefID: 153, Social Networks of Self-care and Perceived Treatment Burden Among Patients on In-Center Hemodialysis<br>Song, M. K.,Paul, S.,Plantinga, L.,Henry, C.,Turberville-Trujillo, L.<br>Level: 2, State: Excluded	Level 2, Form level_2_full_text_screening, This study used the Burden of Treatment Theory: -> None of the above
245	RefID: 245, Minimally Disruptive Diabetes Care for the Elderly<br>Spencer-Bonilla, G.,Rodriguez-Gutierrez, R.,Montori, V. M.<br>Level: 1, State: Excluded	Level 1, Form level_1__titleabstract_screeni
537	RefID: 537, Self-Management in Older Pakistanis Living With Multimorbidity in East London<br>Sultan, Najia,Swinglehurst, Deborah<br>Level: 2, State: Excluded	Level 2, Form level_2_full_text_screening, This study used the Burden of Treatment Theory: -> None of the above
296	RefID: 296, Differences in Time Burden across Local Therapy Strategies for Early-stage Breast Cancer<br>Swanick, Cameron W.,Jiang, Jing,Maldonado, J. Alberto,Lei, Xiudong,Shih, Ya-Chen Tina,Caudle, Abigail S.,Baumann, Donald P.,Giordano, Sharon H.,Shaitelman, Simona F.,Shirvani, Shervin M.,Smith, Benjamin D.<br>Level: 1, State: Excluded	Level 1, Form level_1__titleabstract_screeni
197	RefID: 197, Association Between Number of Preventive Care Guidelines and Preventive Care Utilization by Patients<br>Taksler, G. B.,Pfoh, E. R.,Stange, K. C.,Rothberg, M. B.<br>Level: 1, State: Excluded	Level 1, Form level_1__titleabstract_screeni
233	RefID: 233, “Technology Doesn’t Judge You”: Young Australian Women’s Views on Using the Internet and Smartphones to Address Intimate Partner Violence<br>Tarzia, L.,Iyer, D.,Thrower, E.,Hegarty, K.<br>Level: 2, State: Excluded	Level 2, Form level_2_full_text_screening, This study used the Burden of Treatment Theory: -> None of the above
257	RefID: 257, Limited health literacy in advanced kidney disease<br>Taylor, D. M.,Bradley, J. A.,Bradley, C.,Draper, H.,Johnson, R.,Metcalfe, W.,Oniscu, G.,Robb, M.,Tomson, C.,Watson, C.,Ravanan, R.,Roderick, P.<br>Level: 1, State: Excluded	Level 1, Form level_1__titleabstract_screeni

231	RefID: 231, A systematic review of the prevalence and associations of limited health literacy in CKD<br>Taylor, D. M.,Fraser, S. D. S.,Bradley, J. A.,Bradley, C.,Draper, H.,Metcalfe, W.,Oniscu, G. C.,Tomson, C. R. V.,Ravanan, R.,Roderick, P. J.,Attom Investigators<br>Level: 1, State: Excluded	Level 1, Form level_1__titleabstract_screeni
101	RefID: 101, Toward a Digital Platform for the Self-Management of Noncommunicable Disease: Systematic Review of Platform-Like Interventions<br>Tighe, S. A.,Ball, K.,Kensing, F.,Kayser, L.,Rawstorn, J. C.,Maddison, R.<br>Level: 1, State: Excluded	Level 1, Form level_1__titleabstract_screeni
500	RefID: 500, Patient Priority-Directed Decision Making and Care for Older Adults with Multiple Chronic Conditions<br>Tinetti, Mary E.,Esterson, Jessica,Ferris, Rosie,Posner, Philip,Blaum, Caroline S.<br>Level: 2, State: Excluded	Level 2, Form level_2_full_text_screening, This study used the Burden of Treatment Theory: -> None of the above
501	RefID: 501, Moving From Disease-Centered to Patient Goals-Directed Care for Patients WithMultiple Chronic Conditions Patient Value-Based Care<br>Tinetti, Mary E.,Naik, Aanand D.,Dodson, John A.<br>Level: 1, State: Excluded	Level 1, Form level_1__titleabstract_screeni
260	RefID: 260, Survivorship care and support following treatment for breast cancer: A multi-ethnic comparative qualitative study of women's experiences<br>Tompkins, C.,Scanlon, K.,Scott, E.,Ream, E.,Harding, S.,Armes, J.<br>Level: 2, State: Excluded	Level 2, Form level_2_full_text_screening, This study used the Burden of Treatment Theory: -> None of the above
448	RefID: 448, Rethinking retention: Mapping interactions between multiple factors that influence long-term engagement in HIV care<br>Topp, Stephanie M.,Mwamba, Chanda,Sharma, Anjali,Mukamba, Njekwa,Beres, Laura K.,Geng, Elvin,Holmes, Charles B.,Sikazwe, Izukanji<br>Level: 2, State: Excluded	Level 2, Form level_2_full_text_screening, This study used the Burden of Treatment Theory: -> None of the above
207	RefID: 207, Web-based activity within a sexual health economy: Observational study<br>Turner, K. M. E.,Zienkiewicz, A. K.,Syred, J.,Looker, K. J.,de Sa, J.,Brady, M.,Free,	Level 2, Form level_2_full_text_screening, This study used the Burden of Treatment Theory: -> None of the above

	C.,Holdsworth, G.,Baraitser, P.<br>Level: 2, State: Excluded	
120	RefID: 120, Disconnected relationships between primary care and community-based health and social services and system navigation for older adults: A qualitative descriptive study<br>Valaitis, R.,Cleghorn, L.,Ploeg, J.,Risdon, C.,Mangin, D.,Dolovich, L.,Agarwal, G.,Oliver, D.,Gaber, J.,Chung, H.<br>Level: 2, State: Excluded	Level 2, Form level_2_full_text_screening, This study used the Burden of Treatment Theory: -> None of the above
126	RefID: 126, The feasibility and effectiveness of web-based advance care planning programs: Scoping review<br>van der Smissen, D.,Overbeek, A.,van Dulmen, S.,van Gemert-Pijnen, L.,van der Heide, A.,Rietjens, J. A. C.,Korfage, I. J.<br>Level: 2, State: Excluded	Level 2, Form level_2_full_text_screening, This study used the Burden of Treatment Theory: -> None of the above
247	RefID: 247, Estrogen-progestins and progestins for the management of endometriosis<br>Vercellini, P.,Buggio, L.,Berlanda, N.,Barbara, G.,Somigliana, E.,Bosari, S.<br>Level: 1, State: Excluded	Level 1, Form level_1__titleabstract_screeni
93	RefID: 93, Whose experience is it anyway? Toward a constructive engagement of tensions in patient-centered health care<br>Vogus, T. J.,Gallan, A.,Rathert, C.,El-Manstrly, D.,Strong, A.<br>Level: 2, State: Excluded	Level 2, Form level_2_full_text_screening, This study used the Burden of Treatment Theory: -> None of the above
176	RefID: 176, ‘There’s no pill to help you deal with the guilt and shame’: Contemporary experiences of HIV in the United Kingdom<br>Walker, L.<br>Level: 2, State: Excluded	Level 2, Form level_2_full_text_screening, This study used the Burden of Treatment Theory: -> None of the above
134	RefID: 134, Defining coordinated care for people with rare conditions: A scoping review<br>Walton, H.,Hudson, E.,Simpson, A.,Ramsay, A. I. G.,Kai, J.,Morris, S.,Sutcliffe, A. G.,Fulop, N. J.<br>Level: 2, State: Excluded	Level 2, Form level_2_full_text_screening, This study used the Burden of Treatment Theory: -> None of the above
598	RefID: 598, Developing a taxonomy of care coordination for people living with rare conditions: a qualitative study<br>Walton, H.,Simpson, A.,Ramsay, A. I. G.,Hudson, E.,Hunter, A.,Jones, J.,Ng, P. L.,Leeson-Beevers, K.,Bloom, L.,Kai,	Level 2, Form level_2_full_text_screening, This study used the Burden of Treatment Theory: -> None of the above

	J.,Kerecuk, L.,Kokocinska, M.,Sutcliffe, A. G.,Morris, S.,Fulop, N. J.<br>Level: 2, State: Excluded	
599	RefID: 599, Development of models of care coordination for rare conditions: a qualitative study<br>Walton, H.,Simpson, A.,Ramsay, A. I. G.,Hunter, A.,Jones, J.,Ng, P. L.,Leeson-Beevers, K.,Bloom, L.,Kai, J.,Kokocinska, M.,Sutcliffe, A. G.,Morris, S.,Fulop, N. J.<br>Level: 2, State: Excluded	Level 2, Form level_2_full_text_screening, This study used the Burden of Treatment Theory: -> None of the above
230	RefID: 230, Effect of multifactorial treatment targets and relative importance of hemoglobin A1c, blood pressure, and low-density lipoprotein-cholesterol on cardiovascular diseases in Chinese primary care patients with type 2 diabetes mellitus: A population-based retrospective cohort study<br>Wan, E. Y. F.,Fung, C. S. C.,Yu, E. Y. T.,Chin, W. Y.,Fong, D. Y. T.,Chan, A. K. C.,Lam, C. L. K.<br>Level: 1, State: Excluded	Level 1, Form level_1__titleabstract_screeni
522	RefID: 522, Capturing the experiences of patients across multiple complex interventions: a meta-qualitative approach<br>Webster, Fiona,Christian, Jennifer,Mansfield, Elizabeth,Bhattacharyya, Onil,Hawker, Gillian,Levinson, Wendy,Naglie, Gary,Thuy-Nga, Pham,Rose, Louise,Schull, Michael,Sinha, Samir,Stergiopoulos, Vicky,Upshur, Ross,Wilson, Lynn,Bridges Collaborative<br>Level: 2, State: Excluded	Level 2, Form level_2_full_text_screening, This study used the Burden of Treatment Theory: -> None of the above
117	RefID: 117, An exploration of the experiences of professionals supporting patients approaching the end of life in medicines management at home. A qualitative study<br>Wilson, E.,Caswell, G.,Latif, A.,Anderson, C.,Faull, C.,Pollock, K.<br>Level: 2, State: Excluded	Level 2, Form level_2_full_text_screening, This study used the Burden of Treatment Theory: -> None of the above
535	RefID: 535, The 'work' of managing medications when someone is seriously ill and dying at home: A longitudinal qualitative case study of patient and family perspectives'<br>Wilson, Eleanor,Caswell, Glenys,Pollock, Kristian<br>Level: 2, State: Excluded	Level 2, Form level_2_full_text_screening, This study used the Burden of Treatment Theory: -> None of the above

291	RefID: 291, Postmenopausal women's adherence to pelvic floor muscle exercises over 2 years<br>Wu, C.,Newman, D.,Palmer, M. H.<br>Level: 1, State: Excluded	Level 1, Form level_1__titleabstract_screeni
211	RefID: 211, Communities of clinical practice in action: Doing whatever it takes<br>Young, J.,Jaye, C.,Egan, T.,Williamson, M.,Askerud, A.,Radue, P.,Penese, M.<br>Level: 2, State: Excluded	Level 2, Form level_2_full_text_screening, This study used the Burden of Treatment Theory: -> None of the above
148	RefID: 148, Adaptation and validation of a Spanish version of the treatment burden questionnaire in patients with multiple sclerosis<br>Ysrraelit, M. C.,Fiol, M. P.,Peña, F. V.,Vanotti, S.,Terrasa, S. A.,Tran, V. T.,Montori, V. M.,Correale, J.<br>Level: 1, State: Excluded	Level 1, Form level_1__titleabstract_screeni
308	RefID: 308, Validity and reliability of the Mandarin version of the Treatment Burden Questionnaire among stroke patients in Mainland China<br>Zhang, Qi,Zhang, Ke,Li, Miao,Gu, Jiaxin,Li, Xintong,Li, Mingzi,Jin, Yi<br>Level: 1, State: Excluded	Level 1, Form level_1__titleabstract_screeni
121	RefID: 121, Relational Aspects in Patient-provider Interactions: A Facial Paralysis Case Study<br>Zhu, H.,Moffa, Z. J.,Carroll, J. M.<br>Level: 1, State: Excluded	Level 1, Form level_1__titleabstract_screeni
