# Supplementary material for: A systematic review of the use of burden of treatment theory
Source: J Multimorb Comorb. 2025 May 9;15:26335565251314828. doi: 10.1177/26335565251314828 (PMC12064904; doi:10.1177/26335565251314828)
Supplement: Supplemental Material - A systematic review of the use of burden of treatment theory [file sj-pdf-3-cob-10.1177_26335565251314828.pdf]

Quality appraisal

Relevant adapted CASP checklists used for included papers- if can't answer "yes" to first 2/3 questions, may be of poor-quality evidence.

Appraisal:

CASP checklist :

Brice R. CASP CHECKLISTS - CASP - Critical Appraisal Skills Programme [Internet]. CASP - Critical Appraisal Skills Programme. 2022. Available from: https://casp-uk.net/casp-tools-checklists/

Mixed Studies Review Tool:

Pluye P, Gagnon M, Griffiths F et al. A scoring system for appraising mixed methods research, and concomitantly appraising qualitative, quantitative and mixed methods primary studies in Mixed Studies Reviews. International Journal of Nursing Studies. 2009;46(4):529-546. Available from: https://doi.org/10.1016/j.ijnurstu.2009.01.009

Excel spreadsheet included as part of supplementary materials as evidence of individual paper checklist answers

No exclusion based on quality appraisal of studies. Quality appraisal independently carried out by second and third reviewers using same checklists.

Broad quality scoring category:

All questions answered Yes: Excellent

> 80% : Very good

>50% Yes: Moderate

< 50% Yes: Weak

Overview of study score category for all included papers:

First author	Year	Paper type	Quality appraisal tool used	Quality score
**Abu Dabrh et al [19]**	2021	Qualitative study	CASP checklist	Very good
**Ainsworth et al [20]**	2019	Feasibility trial	CASP checklist	Very good
**Austin et al [21]**	2021	Systematic review	CASP checklist	Excellent
**Brunelli et al [22]**	2021	Case study discussion	N/A	
**Callan et al [23]**	2021	Qualitative study	CASP checklist	Excellent
**Chikumbu et al [14]**	2022	Qualitative study	CASP checklist	Excellent

**Corbett et al [24]**	2020	Qualitative study	CASP checklist	Very good
**Early et al [25]**	2018	Protocol for a mixed methods study	Adapted Mixed Studies Review (MSR) tool	Very good
**Foster et al [26]**	2019	Protocol for a cohort study	CASP checklist	Excellent
**Gilbert et al [27]**	2021	Qualitative study	CASP checklist	Excellent
**Green et al [28]**	2016	Qualitative study	CASP checklist	Very good
**Hounkpatin et al [29]**	2020	Qualitative study	CASP checklist	Excellent
**Hunt et al [30]**	2017	Theory discussion paper	N/A	
**Husebo et al [31]**	2019	Discussion paper	N/A	
**Jakubowski [32]**	2022	Systematic review	CASP checklist	Very good
**Knowles et al [33]**	2017	Qualitative study	CASP checklist	Excellent
**Kyle et al [34]**	2020	Qualitative study	CASP checklist	Very good
**Ladds et al [35]**	2020	Qualitative study	CASP checklist	Excellent
**Lippiett et al [36]**	2018	Systematic review	CASP checklist	Very good
**Lippiett et al [37]**	2022	Qualitative study	CASP checklist	Excellent
**May et al [38]**	2015	Protocol for qualitative meta-synthesis and conceptual modelling study	Adapted CASP checklist	Excellent
**Nordfonn et al [39]**	2019	Qualitative study	CASP checklist	Excellent
**Nordfonn et al [40]**	2020	Qualitative study	CASP checklist	Excellent
**O’Connor et al [41]**	2016	Systematic review	CASP checklist	Excellent
**Papousti et al [42]**	2019	Realist review	CASP checklist	Excellent
**Quigley et al**	2021	Qualitative	CASP	Very good

**[43]**		study	checklist	
**Roberti et al [44]**	2018	Systematic review	CASP checklist	Very good
**Roberti et al [45]**	2021	Qualitative study	CASP checklist	Very good
**Roberti et al [46]**	2022	Qualitative study	CASP checklist	Very good
**Tarzia et al [47]**	2016	Discussion paper	N/A	
