# Supplementary material for: A systematic review of the use of burden of treatment theory
Source: J Multimorb Comorb. 2025 May 9;15:26335565251314828. doi: 10.1177/26335565251314828 (PMC12064904; doi:10.1177/26335565251314828)
Supplement: Supplemental Material - A systematic review of the use of burden of treatment theory [file sj-pdf-4-cob-10.1177_26335565251314828.pdf]

Systematic review of the use of BOTT : search strategy

Initial scoping search undertaken on Google scholar: for articles citing the following citation (the original Burden of Treatment Theory conception paper) :

*May, C.R., Eton, D.T., Boehmer, K. et al. Rethinking the patient: using Burden of Treatment Theory to understand the changing dynamics of illness. BMC Health Serv Res **14**, 281 (2014). https://doi.org/10.1186/1472-6963-14-281*

For the systematic review: citation-based search focusing on the identification of papers which cited the above paper across multiple databases.

Databases searched: Web of Science, Scopus, Medline, CINAHL and medRxiv.org

Searches undertaken: December 2021 and updated June 2022.

Web of Science
Articles citing "rethinking the patient: using Burden of Treatment Theory to understand the changing dynamics of illness": 256 results (June 2022)
Link example of this search strategy used is shown below (Web of Science):
https://www.webofscience.com/wos/woscc/summary/4e1fde1d-1ec2-4e02-b37e-0724fd9f262c-3dc1b071/date-descending/1

Medline via "Ovid"
'Find Citation' tool search:
Articles citing "Rethinking the patient: using Burden of Treatment Theory to understand the changing dynamics of illness" : 72 results (June 2022)

CINAHL (via Ebsco): "Rethinking the patient: using Burden of Treatment Theory to understand the changing dynamics of illness" then 'times cited in this database'
Query: "Rethinking the patient: using Burden of Treatment Theory to understand the changing dynamics of illness" and LINK "Times cited in this database" : 5 results (June 2022)
Interface- EBSCOhost Research Databases
Search screen- advanced search
Database – CINAHL

Scopus :
Search within: References
Search documents: "rethinking the patient: using Burden of Treatment Theory to understand the changing dynamics of illness"
Query: (REF("rethinking the patient: using burden of treatment theory to understand the changing dynamics of illness")) : 274 results (June 2022)

medRxiv.org :
Advanced search: TERM "rethinking the patient: using Burden of Treatment Theory to understand the changing dynamics of illness" : 5 results (June 2022)

The results from these databases were downloaded and screening undertaken on DistillerSR platform. See figures for PRISMA flowchart and Data extraction.

Screening criteria table

Included	Excluded
Original research studies, case studies, systematic reviews, theory or conceptual discussion papers, protocols, conference papers or pre-prints	Editorials, letters, conference abstracts, theses or dissertations
Published in the English language	Passing references to BOTT, did not engage with, apply or discuss the theory
Published after June 2014	
Cited Burden of Treatment Theory conceptual paper [12] and engaged with, applied or discussed the theory, for example:<br>• In data analysis or collection<br>• To guide or inform interview methods<br>• To thematise or characterise data or discussions<br>• To inform methods of intervention development<br>• Any other application	
